# Precision nutrition in diabetic foot ulcers: multimodal artificial intelligence for personalized metabolic management

**DOI:** 10.3389/fnut.2026.1821103

**Published:** 2026-06-17

**Authors:** Hongbin Sun, Xilin Liu, Hong Li

**Affiliations:** 1 Department of Hand and Foot Surgery, China-Japan Union Hospital of Jilin University, Changchun, China; 2 Department of Nursing, China-Japan Union Hospital of Jilin University, Changchun, China

**Keywords:** artificial intelligence, diabetic ulcers, dietary intervention, machine learning, nutritional assessment, personalized nutrition

## Abstract

This review synthesizes AI applications in diabetic foot ulcer (DFU) management, with a particular focus on nutritional and metabolic data integration. Emerging AI methodologies—including image-based dietary assessment, natural language processing-driven chatbots, and continuous glucose monitoring-integrated predictive models—have shown promise in adjacent fields such as general type 2 diabetes management and hemodialysis. However, none have been directly validated in DFU populations, and their applicability to DFU care remains a future research direction rather than a current reality. The main obstacles include the paucity of standardized nutritional data in existing DFU cohorts, methodological barriers in multi-modal data fusion, and the need for robust validation across diverse populations. A future research agenda is proposed, emphasizing the convergence of AI, nutritional science, and multidisciplinary care pathways. By addressing these foundational gaps, AI-enabled approaches may eventually contribute to reducing the global burden of diabetes-related amputations, but substantial methodological and validation work is required before clinical translation can be realistically anticipated.

## 1 Introduction

Diabetic foot ulcers (DFUs) represent a severe, escalating global health burden driven by the increasing worldwide incidence of diabetes. The lifetime prevalence of DFUs ranges from 19 to 34%, and poor clinical management or ulcer progression often results in lower-extremity amputation. Estimates indicate that diabetes-related amputations occur in over one million individuals annually (1). Consequently, the financial burden of diabetic foot care is exceptionally high, accounting for approximately one-third of total diabetes-related healthcare expenditures (2). Beyond the economic costs, DFUs significantly diminish patient quality of life while substantially increasing morbidity and mortality rates (1). Artificial intelligence (AI) may offer substantial potential for improving the diagnosis and treatment of these conditions. Recent deep learning and machine learning methodologies developed for automated DFU assessment have demonstrated high diagnostic accuracy and processing speed. Specifically, a novel convolutional neural network (CNN) architecture, designated Score-DFUNet, achieved an accuracy rate of 95.34% in classifying diverse DFU clinical categories (3).

Advanced AI technology has been developed to assist in the early and rapid diagnosis of DFUs, thereby enabling timely clinical interventions that can mitigate ulcer progression and potentially prevent amputation. Researchers anticipate that this technology will support the creation of highly individualized patient treatment plans by utilizing AI to predict wound healing trajectories and provide highly detailed characterizations of ulcer tissue features (4). Implementing AI within the clinical workflow can optimize routine wound monitoring while enhancing the consistency and objectivity of DFU assessments, which are currently limited by the subjectivity and high variability of non-standardized clinical evaluations (3). Consequently, the purpose of this literature review is to evaluate the current state of the art and explore emerging trends in the application of AI for the clinical management of DFUs. The outcomes and recommendations of this review are expected to contribute to closing existing gaps in current knowledge and clinical research. The overall goal is to equip healthcare professionals to understand and implement available AI technologies for DFU management, thereby enhancing patient outcomes and optimizing healthcare resource utilization (5). Given that DFUs remain a major global health challenge, integrating AI methodologies provides an innovative, efficient approach for early diagnosis and treatment optimization, consequently decreasing the global burden of this severe diabetic complication. Therefore, continuing research at the intersection of AI and diabetic foot care is essential for clinical enhancement and the mitigation of the global DFU epidemic.

## 2 The main causes and risk factors for DFUs

**Figure 1:**
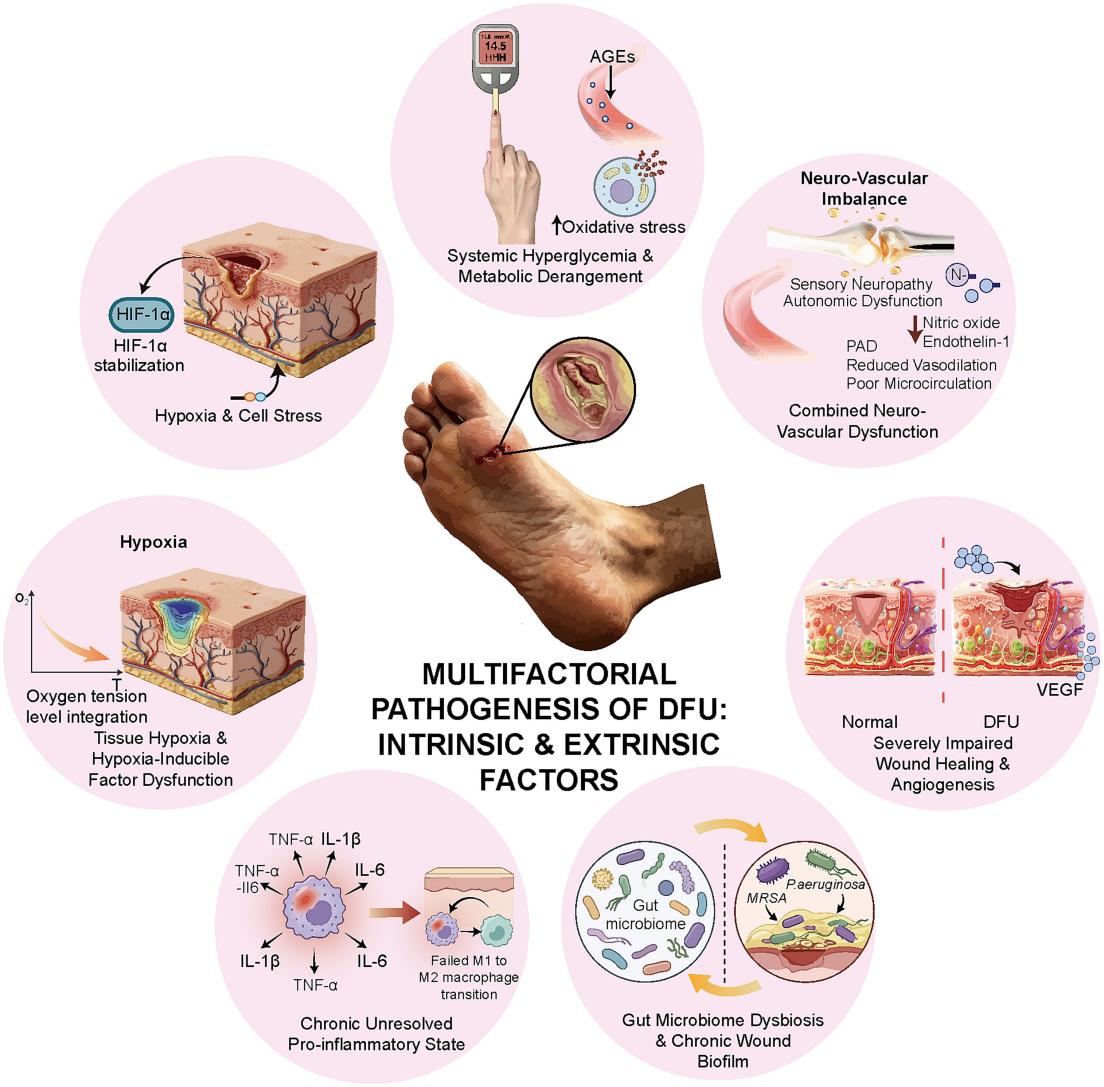
Risk factors for diabetic foot ulcers (DFUs).

DFUs are one of the most common and severe complications of diabetes, imposing a significant clinical and socioeconomic burden worldwide. DFUs develop from a complex interplay between various host and environmental factors (Figure 1).

### 2.1 Nutritional deficiencies

Nutritional status is increasingly recognized as a critical determinant of DFU pathogenesis, healing trajectories, and amputation risk. Nutritional status can be objectively quantified using composite scoring systems derived from routine blood tests, offering a practical and cost-effective approach to risk stratification in clinical wound management.

Two validated nutritional assessment tools have emerged as particularly promising in DFU populations: the Prognostic Nutritional Index (PNI) and the Controlling Nutritional Status (CONUT) score. The PNI is calculated as 10 × serum albumin (g/dL) + 0.005 × total lymphocyte count (per mm^3^), reflecting both nutritional and immunological status. The CONUT score integrates serum albumin, total cholesterol, and total lymphocyte count, thus providing a comprehensive measure of protein reserves, calorie depletion, and immune function (6).

In a retrospective study of 386 patients with DFUs, Coşkun et al. found that PNI values were significantly lower in the amputated patient group than in the non-amputated group (*p* < 0.001). ROC analysis demonstrated that PNI predicted amputation with excellent accuracy (AUC = 0.937, 95% CI: 0.911–0.963); at an optimal cut-off value of 39.005, sensitivity was 82.7%, and specificity was 93.1%. The odds ratio for amputation was 81.8 (95% CI: 38.5–173.7) for patients with PNI values below the cut-off (7). Can et al. subsequently confirmed these findings in a larger cohort of 756 patients, demonstrating that PNI was independently associated with major amputation after multivariable adjustment for age, C-reactive protein (CRP), and erythrocyte sedimentation rate (8). Notably, Oda et al., in a longitudinal cohort study of 349 patients, found that a higher PNI was significantly associated with an increased rate of wound healing. In a multivariable Cox proportional hazards model, each one-standard-deviation increase in PNI yielded a hazard ratio (HR) for wound healing of 1.22. This result was adjusted for multiple confounders, including age, sex, body mass index, hemoglobin A1c, C-reactive protein, low-density lipoprotein cholesterol, history of peripheral artery disease, history of cardiovascular disease, history of amputation, chronic kidney disease, osteomyelitis, use of antihypertensive agents, and smoking status (9).

The association between the CONUT score and DFU severity was similarly validated. In a cohort study of 319 participants (213 healthy controls, 67 diabetic patients without foot ulcers, and 39 DFU patients), Nakamura et al. found that DFU cases had significantly higher CONUT scores than both the control and diabetes-only groups. Within the DFU cohort, a high PEDIS score (>8) was associated with significantly higher CONUT scores, and multivariable logistic regression identified the CONUT score as an independent factor associated with DFU severity (10). Shi et al., in a retrospective study of 357 patients with DFUs, investigated the association between the CONUT score and amputation risk. The authors determined that the total amputation rate was 30.6%, with minor amputations accounting for 98.2% of those cases. Multivariable logistic regression analysis identified moderate-to-severe malnutrition (CONUT score 5–12, OR = 2.685, 95% CI: 1.141–6.314), white blood cell count (OR = 1.091), Wagner grade (OR = 1.872), and ankle-brachial index (ABI, OR = 0.178) as independent risk factors for amputation. The study concluded that the CONUT score is closely associated with amputation risk in DFU patients, indicating that early improvement of nutritional status may reduce such a risk (11).

Among trace elements, zinc plays a particularly important role in wound healing, given its involvement in collagen synthesis, cell proliferation, and immune function. A mini-review by Karakousis et al. reported that most studies show that subjects with DFUs have low serum zinc levels, and there is some evidence that oral zinc supplementation may have a beneficial impact on DFU healing (12). While Karakousis et al. noted that current evidence on zinc and DFUs has not provided definitive answers and more high-quality studies are needed, Nakamura et al. found that serum zinc levels were significantly lower in DFU patients compared to controls, and multivariable analysis identified lower serum zinc levels as an independent factor associated with wound healing failure (10). Valayatham et al. similarly showed that among patients in the lowest tertile of serum zinc (<13 μmol/L), the mean healing rate at week 12 was significantly slower than that of patients in the second tertile (13–14 μmol/L) (18.9 vs. 38.0 mm^2^/week, *p* = 0.04). A critical unresolved issue raised by the authors is whether low serum zinc represents a causal mediator of poor DFU outcomes or merely a disease marker that co-occurs with comorbidities. Notably, after adjusting for age, serum albumin, serum creatinine, known ischemic heart disease, and initial ulcer area, baseline zinc tertiles were no longer significantly associated with final healing rates, suggesting that zinc levels may be confounded by comorbidity rather than exerting a direct independent effect on wound healing (13). After a decade of subsequent research, no randomized controlled trial (RCT) to date has definitively separated zinc deficiency as an independent causal factor from the residual confounding inherent in observational studies. Importantly, normalizing zinc levels through supplementation does not necessarily reverse the wound healing trajectory if zinc deficiency is a surrogate for broader nutritional deterioration—a phenomenon reminiscent of the “albumin paradox” where hypoalbuminemia predicts poor outcomes but albumin infusion fails to improve them. Pragmatically, while zinc assessment remains clinically useful for identifying high-risk patients, the therapeutic decision for supplementation should rely on RCT evidence rather than observational associations alone. This underscores that, despite decades of investigation, it remains unresolved whether zinc deficiency is a marker or a mediator of DFU pathology—a distinction with direct implications for future intervention trial design and clinical practice guidelines.

Beyond zinc, vitamin D deficiency has gained recognition as another significant nutritional concern in DFU patients. A comprehensive meta-analysis by Tang et al., incorporating 36 studies and 11,298 individuals, demonstrated that there is a significant dose–response association between vitamin D deficiency or insufficiency and an elevated risk of DFU occurrence, with odds ratios of 3.28 for vitamin D levels <25 nmol/L, 2.25 for levels <50 nmol/L, and 1.67 for levels <75 nmol/L (14). A subsequent umbrella review by Liu et al., incorporating eight meta-analyses, confirmed that patients with DFUs have significantly lower serum vitamin D levels than those without, with differences ranging from −7.14 to −0.93 ng/mL. The study further indicated that individuals with severe vitamin D deficiency have at least an 1.82-fold greater susceptibility of developing DFUs (15). However, caution is warranted when interpreting associations between vitamin D and DFUs due to the potential bias present in the included studies. Many are observational and subject to residual confounding, which precludes definitive causal inference.

#### 2.1.1 Other micronutrients and protein–energy malnutrition

Deficiencies in multiple micronutrients, including vitamin A, vitamin C, selenium, and iron, have been reported in patients with DFUs, although the number of studies for each nutrient is limited. The systematic review by Apergi et al., which included 41 studies, underscored that malnutrition and micronutrient deficiencies are highly prevalent in patients with DFUs and are generally associated with adverse outcomes, including delayed wound healing, higher amputation rates, and increased mortality (16). In a five-year retrospective study, Yan et al. found that protein malnutrition, as reflected by low serum albumin (<30 g/L), was associated with significantly higher amputation rates (31.5% vs. 8.8%) and all-cause mortality rates (45.8% vs. 17.1%) compared with patients maintaining normal albumin levels (>30 g/L). Multivariable logistic regression further identified hypoalbuminemia as an independent risk factor for all-cause mortality (OR = 3.38, 95% CI: 1.40–8.18, *p* = 0.007), but not for amputation (*p* = 0.128) (17). These findings collectively highlight that comprehensive nutritional assessment—incorporating both composite scores (PNI, CONUT) and targeted micronutrient measurements (zinc, vitamin D, albumin)—is essential for accurate risk stratification and should inform the development of personalized nutritional interventions in DFU care.

#### 2.1.2 Implications for AI-enabled precision nutrition

The robust associations between these nutritional parameters and DFU outcomes establish a strong foundation for integrating nutritional data into AI-driven predictive models. PNI and CONUT scores, being derived from routinely available laboratory parameters, are highly compatible with machine learning applications. Similarly, the dose–response relationships observed for vitamin D and zinc suggest that these continuous biomarkers could serve as valuable inputs for AI-based risk calculators. Furthermore, the heterogeneity in patient nutritional status underscores the need for personalized—rather than standardized—nutritional interventions, a task well-suited for machine learning algorithms capable of integrating multimodal data, including laboratory results, dietary patterns, and clinical trajectories. However, caution is warranted when interpreting these associations. A recent systematic review and meta-analysis by Donnelly et al., which synthesized 23 studies on nutritional interventions in diabetes-related foot ulcer populations and employed GRADE to assess the certainty of evidence, concluded that the evidence relating to the wound-healing-ameliorating effects of nutrient supplementation remains of low to very low certainty (18). Specifically, although meta-analyses showed that nutrient supplements significantly reduced wound depth [WMD –0.200 mm (95% CI –0.364 to −0.035)], width [WMD –0.466 mm (−0.724 to −0.208)], and length [WMD –0.443 mm (−0.841 to −0.045)], the quality of the included studies was low and there was substantial between-study heterogeneity (*I*^2^ = 56–68%). This very low certainty of evidence—reflecting serious concerns regarding risk of bias, inconsistency, and imprecision—indicates that the current body of nutritional research in DFU remains largely observational and underpowered for causal inference. Consequently, although the associations described above are clinically compelling, they should be interpreted as hypothesis-generating rather than definitive, underscoring the need for well-designed RCTs powered to evaluate cause-and-effect relationships between nutritional parameters and DFU outcomes. This limitation is further reflected in recent prospective data; for instance, Hulshagen et al. conducted a prospective observational cohort study of 78 hospitalized patients with DUCs and found that patients identified as malnourished at admission exhibited significantly lower baseline skeletal muscle mass than non-malnourished patients, whereas baseline handgrip strength did not differ significantly between the groups During hospitalization, no significant changes in muscle mass or strength were observed, regardless of nutritional status at admission, suggesting that standard clinical care, including nutritional support, may stabilize muscle health in this population (19). Notably, in malnourished patients, the standard nutritional support provided during hospitalization prevented a further decline in muscle mass and muscle function. These data indicate that early and adequate nutritional intervention plays a critical role in preserving muscle mass in malnourished patients, thereby mitigating muscle atrophy and associated clinical complications. In summary, these findings suggest that structured nutritional interventions are necessary to promote wound healing and prevent adverse outcomes, such as amputation, in patients with DFUs. This objective is achieved by optimizing micronutrient levels, including zinc, and improving baseline nutritional status to achieve optimal PNI and CONUT scores (Table 1).

**Table 1:** Nutritional and metabolic parameters for DFU prognosis and AI-driven nutrition.

Category	Parameter metric	Key findings/prognostic relevance	References
Nutritional and inflammatory biomarkers	Prognostic nutritional index (PNI)	In DFU patients, a low PNI (≤39.005) independently predicted amputation with excellent accuracy (AUC = 0.937; OR = 81.8), showing a sensitivity of 82.7% and specificity of 93.1%.	(7)
Nutritional and inflammatory biomarkers	Prognostic nutritional index (PNI)	In DFU patients, a lower PNI was independently associated with an increased risk of sepsis (AUC = 0.702; optimal cut-off ≤34.75), with a sensitivity of 56.0% and specificity of 77.7%.	(129)
Nutritional biomarker	Controlling nutritional status (CONUT) score	A high CONUT score was independently associated with greater ulcer severity (high PEDIS score, OR = 2.10, *p* = 0.0206) but showed no significant association with wound healing failure (*p* = 0.0613) in DFU patients.	(10)
Nutritional biomarker	Serum Zinc	In DFU patients, low serum zinc level was independently associated with wound healing failure (OR = 0.794, *p* = 0.00556), with significantly lower zinc levels in the failure group compared to the success group (47.6 vs. 62.8 μg/dL, *p* < 0.001).	(10)
Nutritional biomarker	Serum albumin	Albumin predicts short-term DFU healing only when below normal; changes within the normal range have no predictive value.	(130)
Nutritional biomarker	Zinc	Serum zinc levels are lower in patients with diabetic foot ulcers compared to diabetics without foot ulcers, with more severe ulcers linked to greater zinc deficiency. Zinc supplementation at 50 mg/day for 12 weeks reduces ulcer size, whereas zinc deficiency compromises antioxidant defenses and delays healing.	(36)
Nutritional biomarker	Vitamin D	Taking 2000 IU/day of vitamin D for 12 weeks cut infection rate from 45 to 25%, reduced severe infections needing antibiotics (10% vs. 25%), shrank ulcers by 60% versus 35%, raised 25(OH)D from 16.5 to 35.2 ng/mL, boosted cathelicidin by 30%, lowered IL-6 and TNF-*α* by 20%, and caused no hypercalcemia.	(36, 131)
Glycemic control	HbA1c	HbA1c ≥ 8% raised the odds of lower-extremity amputation (pooled OR = 5.43) and fasting glucose ≥126 mg/dL also increased amputation risk (OR = 1.46), but HbA1c > 7.0–7.5% did not, and HbA1c was not linked to wound healing (OR = 0.44; HR = 1.01).	(132)
Glycemic control	Fasting glucose	A fasting glucose of 126 mg/dL or above was linked to a higher risk of lower-limb amputation (pooled OR = 1.46, 95% CI 1.02–2.09).	(132)
Data type for AI modeling	Dietary intake records	The Xception model classified high-GI foods (rice, congee) and low-GI milk with 100% train/test accuracy; using 25 depth points, MLP models predicted leftover rice (RMSE 18.28, MAPE 3.13%), congee (RMSE 1.42, MAPE 0.60%), and milk (RMSE 0.57, MAPE 0.30%), outperforming linear regression (rice MAPE 3.13% vs. 9.17%) and six dietitians (39–47% MAPE).	(133)
Body composition analysis	Phase angle	Lower phase angle was independently linked to worse DFU healing: each 1° rise in PhA cut the 6-month non-healing risk by 20% (RR = 0.80), the best cutoff was 3.8° (AUC = 0.699), and PhA ≤ 3.8° raised non-healing risk by 37% (RR = 1.37); the effect was stronger in women (RR = 5.40) and those with eGFR ≥60 (RR = 2.01); PhA correlated positively with nutritional markers (*r* = 0.50–0.55) and negatively with hydration (*r* = −0.77), so low PhA points to malnutrition, poor cell integrity, and higher cardiovascular risk.	(134)
Micronutrient levels	Vitamin D, vitamin C, vitamin A, zinc, magnesium, selenium, copper, ferritin, folate, vitamin B-12	Vitamin D deficiency was most common (55–87% of DFU cases) and linked to higher pro-inflammatory cytokines; vitamin D supplementation (300,000 IU once or 2000 IU/day) improved healing (OR = 4.11); vitamin C deficiency occurred in 42–59%, and 500 mg/day sped healing (50% healing time 20 vs. 48 days); high-dose folate (mean 192 ng/mL) gave 90% complete healing; zinc (50 mg/day elemental for 12 weeks) cut ulcer length and width (*p* = 0.02); magnesium plus vitamin E also reduced ulcer size (*p* = 0.003–0.02); raised ferritin marked more severe foot disease (*p* = 0.004) and inflammation.	(36)

In addition to nutritional and metabolic influences, DFU development is driven by a complex interplay among other clinical risk factors, including peripheral neuropathy, peripheral vascular disease, structural foot deformities, deep tissue infection, and mechanical stressors such as inappropriate footwear or inadequate foot care practices. These non-nutritional dimensions have been extensively reviewed elsewhere, and they are detailed in Supplementary file 1.

### 2.2 Poor glycemic control

Poor glycemic control has long been recognized as a major contributor to the risk of diabetes complications, and its specific role in DFU pathogenesis, progression, and prognosis has been substantiated by a large body of evidence. In a retrospective cohort study of 1,876 patients with type 2 diabetes, Zheng et al. (20) demonstrated a distinct dose–response relationship between long-term glycemic burden and DFU incidence. As the mean cumulative glycemic burden (MCGB) increased across quartiles, DFU incidence rose from 3.2% in the lowest quartile to 16.0% in the highest quartile. Notably, multivariate Cox regression analysis revealed that both chronic hyperglycemia and glycemic fluctuations independently predicted DFU risk; patients in the highest quartiles for MCGB and variability cumulative glycemic burden (VCGB) exhibited significantly elevated risks, with hazard ratios of 2.99 and 5.29, respectively (20). These data indicate that minimizing both overall average glucose levels and acute glycemic volatility is critical for effective DFU prevention. A comprehensive systematic review and meta-analysis by Dutta et al. (21), which included 47 observational studies comprising 12,604 patients with DFUs, evaluated the association between glycemic control and DFU outcomes. Compared with HbA1c levels below 8%, an HbA1c of 8% or higher was associated with a significantly increased risk of lower extremity amputation, with a pooled odds ratio of 4.80 (95% CI: 2.83–8.13). Similarly, fasting glucose levels of 126 mg/dL or greater conferred a pooled OR for LEA of 1.46 (95% CI: 1.02–2.09). These findings indicate that poor glycemic control, reflected by both elevated HbA1c and fasting glucose, is a substantial risk factor for amputation in patients with DFUs. In a prospective nested cohort study of 43 patients with poorly controlled diabetes and neuropathic DFUs, Dutta et al. found that individuals whose ulcers healed within 12 weeks had significantly lower HbA1c levels at 4 weeks (7.7% vs. 8.9%, *p* = 0.001) and at 12 weeks (6.8% vs. 7.6%, *p* = 0.018) compared with those whose ulcers did not heal. Cox regression analysis identified an HbA1c level at 4 weeks >8.15% as a significant predictor of delayed DFU healing. The authors concluded that early and intensive glycemic control within the first 4 weeks of treatment is associated with greater healing, independent of initial ulcer area (21).

#### 2.2.1 Glycemic variability

It is increasingly recognized that glycemic variability (GV)—the extent of day-to-day or visit-to-visit glucose fluctuations—may exert harmful effects on DFU outcomes independent of mean HbA1c. In a retrospective study of 300 individuals with type 2 diabetes and DFUs, Caruso et al. (22) stratified participants according to their glucose coefficient of variation (CV), using a threshold of 36%. The high GV group (≥36%) had significantly longer diabetes duration, higher HbA1c, and higher urinary albumin excretion than the low GV group (<36%). Multiple logistic regression analysis identified both the CV and the standard deviation of glucose as independent predictors of healing failure. Furthermore, individuals in the high GV cohort exhibited a three-fold higher risk of healing failure within 6 months compared to those with stable glucose levels (22). These findings suggest that reducing glycemic fluctuation may represent an important therapeutic target alongside lowering mean HbA1c.

#### 2.2.2 HbA1c variability and DFU healing

The relationship between long-term HbA1c variability and DFU healing outcomes has also been examined. In a retrospective analysis of the UK National Diabetic Foot Care Audit (NDFA) data, Thomason et al. (23) examined the association between HbA1c variability and DFU healing. After adjusting for ulcer characteristics, ischemia, and diabetes duration, an HbA1c variability of 6–10 mmol/mol (vs. <6 mmol/mol) was associated with an adjusted odds ratio of 1.76 (95% CI: 1.1–2.8) for presenting with an active ulcer at 12 weeks. However, at 12 months, only ischemia (aOR: 2.4) and a diabetes duration >24 years (aOR: 3.3) remained significant predictors of healing, whereas ulcer site, baseline area, and HbA1c variability did not. The authors concluded that low GV is associated with a greater odds of healing in the short term, but that poor blood flow and longer diabetes duration remain the strongest predictors of poor long-term outcomes (23).

#### 2.2.3 Continuous glucose monitoring-derived metrics

Technological advances in continuous glucose monitoring (CGM) have enabled more precise characterization of glycemic control beyond HbA1c. Because even minor hyperglycemia can impair wound healing, maintaining glucose stability may be as crucial as lowering average glucose. Initially, a pilot study by Srinivasan et al. (24) of 22 patients with type 2 diabetes mellitus revealed that the use of CGM was associated with both significant improvements in glycemic control and positive influences on wound healing. Extending these findings, a subsequent prospective study by Ortiz-Zúñiga et al. (25) investigated the mechanistic link between specific CGM metrics and ulcer closure timelines. They observed that Time in Range (TIR) was inversely correlated with the elapsed time to complete ulcer closure. Conversely, both Time Above Range (TAR) and the Glucose Management Indicator (GMI) were directly correlated with a prolonged healing process (*p* < 0.05). These results indicate that real-time glycemic parameters captured *via* CGM are tightly coupled with the rate of clinical wound healing (25). Furthermore, a larger study within a safety-net health system demonstrated that, at 3–4 months, 72% of patients undergoing CGM achieved fully healed foot wounds compared to only 47% in a pre-intervention cohort, despite a similar baseline prevalence of peripheral arterial disease.

#### 2.2.4 Mechanistic basis

The molecular mechanisms underpinning the detrimental effects of poor glycemic control on DFU outcomes are well-established. Persistent hyperglycemia triggers oxidative stress, chronic inflammation, and the accumulation of advanced glycation end-products (AGEs), which together disrupt sequential stages of wound healing—including immune cell recruitment, angiogenesis, and extracellular matrix remodeling. These aberrations drive the characteristic non-healing phenotype of DFUs.

#### 2.2.5 Poor glycemic control and DFU recurrence

Beyond initial DFU development and healing, poor glycemic control has been identified as a significant predictor of DFU recurrence. In a prospective study of DFU recurrence (Eurodiale subgroup, *n* = 73, 3-year follow-up), the recurrence rate was 57.5%. Independent predictors were plantar ulcer location (OR = 8.62), osteomyelitis (OR = 5.17), HbA1c > 7.5% (OR = 4.07), and CRP > 5 mg/L (OR = 4.27), with plantar location being the strongest (26). The Fukuoka Diabetes Registry, a large prospective cohort study, further confirmed that poor glycemic control, along with a history of DFU, chronic kidney disease, and depressive symptoms, was a significant risk factor for developing foot ulcers (27). Notably, participants who developed a DFU had significantly lower 5-year survival rates compared with those without a DFU (87.7% vs. 95.3%).

#### 2.2.6 Implications for AI-enabled precision nutrition and glycemic management

There is robust and multifaceted evidence linking glycemic control to DFU outcomes, providing a strong foundation for integrating glycemic parameters into AI-driven predictive models and personalized intervention strategies. Unlike conventional clinical risk calculators that rely on a single HbA1c value, AI architectures can incorporate longitudinal glycemic trajectories, CGM-derived metrics (including TIR and GV indices), and dynamic lifestyle data to generate real-time risk stratification and provide adaptive treatment recommendations. The demonstration that early intensive glycemic control within the first 4 weeks of treatment significantly accelerates early wound area reduction underscores the importance of timely metabolic intervention—a goal that AI-enabled continuous monitoring and personalized dietary algorithms are uniquely positioned to achieve. Furthermore, the distinct contributions of mean glucose levels and GV to DFU outcomes suggest that AI models designed to optimize both dimensions of glycemic control may yield superior clinical benefits compared to conventional approaches that target HbA1c alone.

#### 2.2.7 Neurological and biomechanical risk factors

**Figure 2:**
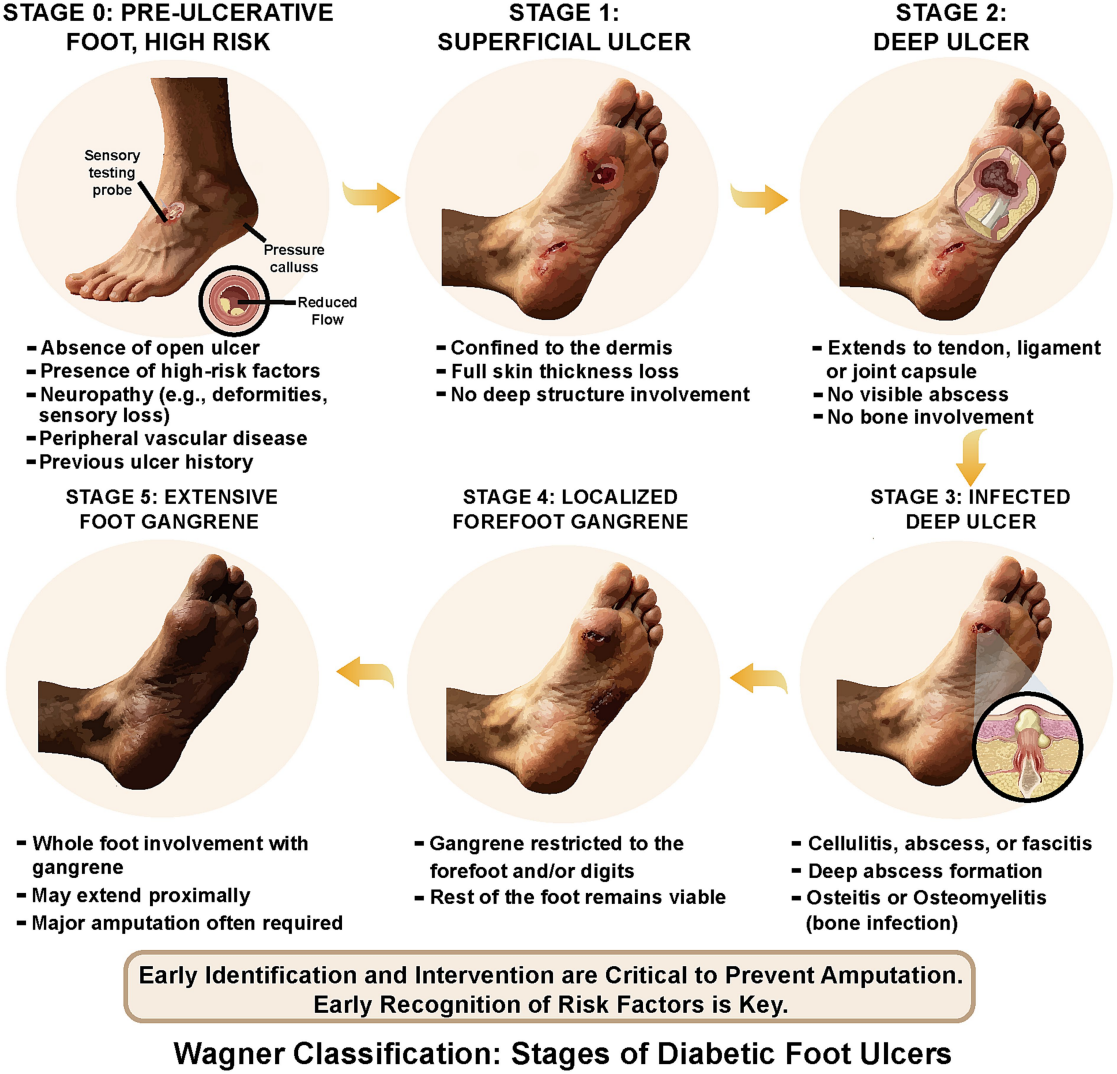
Presentation of different stages of diabetic foot ulcer (DFU) development.

Beyond nutritional and metabolic factors, multiple clinical dimensions contribute to DFU development, delayed healing, and poor prognosis. Diabetic peripheral neuropathy (DPN) affects up to 50% of individuals with long-standing diabetes and represents the single most important risk factor for foot ulceration; a meta-analysis including 20 studies and 4,238 patients identified DPN as a significant predictor of DFU recurrence, with an odds ratio of 4.05 (28). Concurrently, foot deformities in combination with DPN substantially alter plantar pressure distribution, and a systematic review confirmed that elevated barefoot plantar pressure is associated with a higher risk of ulcer development (29) (Figure 2).

## 3 The prevention of DFUs

Because DFUs can lead to severe complications, including infections and amputations, establishing a preventive approach is crucial for patients with diabetes.

### 3.1 Nutritional support

Poor nutritional status—encompassing protein-energy malnutrition, specific vitamin and trace element deficiencies, and altered body composition—is highly prevalent among patients with DFUs. This systemic depletion has been consistently associated with delayed wound healing, an increased risk of amputation, and elevated mortality. Recent systematic reviews, meta-analyses, and RCTs have highlighted the importance of nutritional support for DFU management (30).

Protein-energy malnutrition is a key modifiable risk factor in patients with DFUs. In a double-blind RCT by Eneroth et al., 53 patients with a DFU received either a 400 kcal oral nutritional supplement or a placebo for 6 months. No statistically significant difference in healing rates was observed between the two groups (31). A subsequent prospective RCT of 270 patients by Armstrong et al. examined a nutritional supplement containing arginine, glutamine, and *β*-hydroxy-β-methylbutyrate (HMB) (32). While overall DFU healing rates did not differ between the supplementation and control groups at 16 weeks, post-hoc analysis revealed encouraging findings in high-risk subgroups. Among patients with serum albumin levels ≤40 g/L, 46.8% in the supplementation group healed compared with 30.2% in the control group (*p* = 0.03), while among those with an ankle-brachial index <1.0, the healing rates were 38.0% vs. 23.7% (*p* = 0.008). The authors concluded that the addition of arginine, glutamine, and HMB to standard care may improve DFU healing specifically in patients at risk of poor limb perfusion, low albumin levels, or both.

A recent systematic review and meta-analysis by Donnelly et al., which included 23 studies evaluating diabetes-related foot ulcer populations, investigated the effectiveness of dietary interventions (18). They found that nutrient supplements significantly reduced wound depth, width, and length. However, the quality of the included studies was low, substantial heterogeneity was detected, and the certainty of the evidence was rated as very low. The authors concluded that evidence supporting nutrient supplementation for wound healing remains weak, though nutrition education may help reduce wound size.

Zinc plays a critical role in wound healing through its involvement in collagen synthesis, cell proliferation, and immune function. Compared with a placebo, zinc supplementation has been associated with significantly larger reductions in ulcer length and width. Zinc supplementation was also reported to improve glycemic and metabolic parameters, including fasting plasma glucose, serum insulin, HOMA-IR, HbA1c, and serum HDL-cholesterol. However, these findings are subject to an Expression of Concern regarding data reliability (33). Separately, a systematic review by Batarbekova et al. summarizing data on micronutrient status in DFU patients found that elevated concentrations of folate and vitamin B-12 were associated with improved ulcer healing, and that supplementation with zinc and magnesium contributed to a reduction in ulcer size (34).

The relationship between vitamin D and DFU outcomes has been extensively investigated. An umbrella review of eight meta-analyses by Liu et al. confirmed that individuals with DFUs exhibit significantly lower serum vitamin D levels than patients with diabetes without DFUs, and that severe vitamin D deficiency is associated with a 1.82 times greater susceptibility to developing an ulcer (15). Additionally, vitamin D supplementation has been shown to reduce both the size and number of ulcers in this patient population. A meta-analysis of seven RCTs involving 580 patients by Wu et al. demonstrated that vitamin D supplementation significantly promotes DFU healing, specifically improving the wound healing efficiency rate, accelerating the reduction in wound area, and elevating serum 25(OH)D levels (35). This intervention also improved glucose metabolism and reduced inflammatory marker levels.

Beyond zinc and vitamin D, deficiencies in selenium, vitamins C, E, and magnesium have been reported in DFU populations. A systematic review by Apergi et al. emphasized the need for individualized correction of vitamin D, C, E, and selenium deficiencies, noting that a Mediterranean diet may benefit wound healing (16). Similarly, Batarbekova et al. reported that supplementation with zinc and magnesium contributed to a reduction in ulcer size (36). Several RCTs have also investigated the effects of magnesium and vitamin E co-supplementation, as well as omega-3 PUFA supplementation from flaxseed oil, on wound healing and metabolic profiles in patients with DFUs, yielding encouraging though inconclusive results.

A combined approach using nutrient-dense supplementation together with structured nutrition education appears to be particularly promising. Basiri et al. reported that, in addition to faster wound healing, the combination of nutritional supplementation and education led to a significant decrease in plasma IL-6 concentration, indicating that the intervention exerts an anti-inflammatory effect (37, 38).

Dietary patterns rather than isolated nutrients may offer broader benefits for DFU prevention and management. The Mediterranean diet, characterized by high intakes of fruits, vegetables, whole grains, legumes, nuts, olive oil, and moderate fish consumption, has been associated with reduced inflammation and improved glycemic control. A narrative review by Yu et al. highlighted that combining machine learning analytics with CGM enables real-time adjustment of macronutrient ratios, thereby improving individualized care (39). Disturbances in nutrient metabolism—including impaired amino acid and lipid handling—are key drivers of diabetic neuropathy and foot ulceration, making AI-guided precision nutrition a logical next step in DFU management.

For patients receiving enteral or parenteral nutrition, standard formulas may be insufficient to meet the elevated protein and micronutrient demands associated with wound healing. The Wound Healing Society recommends an energy intake of 30–35 kcal/kg per day and a protein intake of 1.25–1.5 g/kg per day for patients with chronic wounds who are at risk of malnutrition. In clinical practice, specialized nutritional formulas enriched with arginine, glutamine, HMB, zinc, selenium, and antioxidants are frequently used to support tissue synthesis and wound repair.

Despite the growing body of evidence, the overall quality of studies on nutritional support in DFU care remains low to moderate, with substantial heterogeneity in study design, outcome measures, and patient populations. The systematic review by Donnelly et al. noted that the certainty of evidence supporting nutrient supplementation in DFUs is very low, and that a lack of consistency in wound healing measurement and a lack of diet quality assessment represent major methodological gaps (18). Future research should prioritize the design of robust RCTs featuring standardized nutritional phenotyping, longer follow-up periods, and the integration of dietary pattern assessments alongside isolated nutrient supplementation. Furthermore, translating these nutritional principles into AI-enabled precision nutrition tools—such as personalized dietary recommendation systems, real-time nutrient tracking *via* image recognition, and machine learning models that integrate laboratory biomarkers, dietary intake, and wound healing trajectories—represents a promising but largely unexplored frontier in DFU care. The heterogeneity of patient nutritional status, the dose–response relationships observed for several micronutrients, and the potential for nutrient-drug interactions all underscore that personalized rather than one-size-fits-all nutritional interventions are required. This is a clinical challenge that machine learning is uniquely positioned to address, involving the simultaneous tracking of laboratory parameters, wound characteristics, dietary patterns, and longitudinal clinical outcomes.

### 3.2 Control of blood sugar levels

Maintaining blood glucose levels within target ranges is critical for preventing DFU development and promoting ulcer healing in patients who already have foot ulcers. Regular monitoring and management of diabetes through diet, exercise, and medication help maintain optimal blood glucose levels, thereby decreasing the risk of DFUs. Abuhaq et al. demonstrated that the incidence of DFUs is associated with changes in fasting blood glucose (FBG) among patients with diabetes mellitus, emphasizing the importance of close blood glucose monitoring for reducing the risk of ulceration (40). A multicenter cross-sectional study conducted by Vahwere et al. in Uganda showed that most patients with DFUs (76.9%) had poor glycemic control, with 81.4% of those in the severe DFU group exhibiting poor glycemic control. However, multivariate analysis did not find a significant association between the level of glycemic control and DFU severity. Instead, independent factors associated with severe DFUs identified in the study included an age of 70–95 years, mild to moderate neuropathy, and an ulcer diameter greater than 5 cm. Conversely, primary and secondary education levels, moderate to severe visual impairment, and regular vegetable consumption were associated with lower DFU severity (41). Ullas et al., in a descriptive correlational study of hospitalized patients with DFUs (*n* = 159), found that the total Diabetes Distress Scale score was not significantly correlated with glycated hemoglobin (HbA1c), FBG, or random blood glucose (RBG). However, the emotional burden subscale showed a significant negative correlation with FBS. Additionally, FBS was positively correlated with HbA1c. The study suggests that the relationship between diabetes-related distress and glycemic indicators is complex and requires further differentiation of the distinct dimensions of psychological distress (42).

The clinical importance of glycemic control in DFU management is supported by trial evidence. A meta-analysis of nine RCTs (*n* = 10,897) found that intensive glycemic control was associated with a 35% reduction in amputation risk (43). Long-term follow-up of the DCCT/EDIC trial (*n* = 1,441, type 1 diabetes) showed that early intensive control reduced DFU risk by 23% over 23 years (44). These findings support the IWGDF 2023 guideline recommendation that glycemic control should be optimized as part of comprehensive DFU management (45). Nutritional interventions designed for glycemic optimization are essential for both DFU prevention and wound healing. Donnelly et al. conducted a systematic review and meta-analysis that included 23 studies of populations with diabetes-related foot ulcers to evaluate the effects of dietary strategies on chronic wound healing. Their results showed that nutrient supplements significantly reduced wound depth, width, and length (18). Among the two studies that evaluated nutrition education, one reported a significant reduction in wound area, and the other a significant increase in the proportion of participants achieving complete healing. The authors concluded that nutrient supplementation may promote wound healing and that nutrition education may play a role in reducing wound size; however, the quality of the available evidence remains low, and further high-quality RCTs are needed to validate these conclusions.

These findings align with the broader principle that optimizing nutritional status is a prerequisite for achieving glycemic targets. In practice, dietary interventions for patients with DFUs should prioritize carbohydrate consistency to minimize postprandial glucose excursions, an adequate protein intake equal to or exceeding 1.2–1.5 g/kg per day to support wound repair without compromising glycemic control, the incorporation of low-glycemic-index foods to improve insulin sensitivity, and the avoidance of energy-dense, nutrient-poor foods that exacerbate hyperglycemia and systemic inflammation. The role of registered dietitians or certified diabetes educators in delivering individualized medical nutrition therapy has been shown to improve both glycemic metrics and DFU healing outcomes. CGM represents a significant technological advancement in real-time intervention, markedly improving the ability to achieve and maintain glycemic control in patients with DFUs. A pilot study by Srinivasan et al. retrospectively evaluated 22 patients with type 2 diabetes and active DFUs who were provided with CGM devices as part of their care. The mean HbA1c prior to CGM introduction was 84.10 mmol/mol (range 54–132). After 3 months of CGM use, the mean HbA1c was reduced to 65.05 mmol/mol (range 32–94), representing a mean reduction of 19.05 ± 22.07 mmol/mol, with the greatest improvements observed in patients who had higher baseline HbA1c levels. Wound size decreased from a baseline median of 1.53 cm^2^ (IQR 0.75–7.62) to 0.42 cm^2^ (IQR 0.0–1.16) after 3 months (*p* < 0.001), and three patients achieved complete wound healing. It was concluded that the use of CGM significantly improves glycemic control and has a positive influence on wound healing (24).

The relationship between CGM-derived metrics and DFU healing has been further elaborated in a prospective study by Ortiz-Zúñiga et al. Patients with type 2 diabetes and non-complicated DFUs were fitted with a CGM device until ulcer closure. An inverse correlation was observed between the time to achieve complete ulcer closure and TIR (the percentage of time glucose remains within the target range of 70–180 mg/dL; *p* = 0.005). In addition, direct correlations were found between healing time and both TAR and the GMI (*p* < 0.05). These findings suggested that glycemic control is directly related to the healing of non-complicated DFUs (26) and substantiate the clinical value of real-time CGM data in guiding dietary and pharmacologic adjustments to promote DFU healing.

#### 3.2.1 Medication considerations in the management of glycemia in DFU patients

Pharmacological optimization of glycemic control requires careful attention to medication safety and wound healing. Metformin, with its established cardiovascular safety profile and effects on insulin sensitivity, remains a first-line oral agent in patients with DFUs who do not present with contraindications, such as advanced chronic kidney disease or acute ischemic tissue compromise. Sodium-glucose cotransporter-2 (SGLT2) inhibitors and glucagon-like peptide-1 (GLP-1) receptor agonists have become valuable adjuncts given their demonstrated cardiovascular and renal benefits. However, SGLT2 inhibitors require close clinical monitoring in patients with active DFUs or those at high risk for lower-extremity complications. A nationwide cohort study from Denmark reported that the risk of lower limb amputations was increased with SGLT2 inhibitor use compared with sulfonylureas, although these findings remain contentious and may reflect channeling bias. Insulin therapy is often preferred in hospitalized patients with DFUs who require rapid glycemic stabilization and dose titration. Conversely, sulfonylureas and thiazolidinediones are generally avoided due to risks of hypoglycemia and fluid retention, respectively.

#### 3.2.2 Integration with AI-enabled nutritional support

The translation of glycemic management principles into AI-enabled precision nutrition tools represents a promising yet largely unexplored frontier in DFU care. Unlike conventional dietary counseling based on static guidelines, AI architectures can integrate real-time CGM data, dietary intake records, and wound healing trajectories to generate personalized, just-in-time nutritional recommendations. For example, machine learning models trained on CGM data can predict postprandial glycemic responses to specific food combinations, enabling individualized meal planning that minimizes glucose fluctuations while meeting the increased protein and micronutrient demands associated with wound healing. The evidence that TIR, rather than a single HbA1c value, is closely correlated with healing time—and that a TIR of less than 70% is an independent predictor of delayed wound closure—underscores the potential of AI-driven systems that optimize glycemic metrics as dynamic therapeutic targets. Furthermore, AI-enabled image recognition apps for dietary assessment can provide patients and clinicians with real-time feedback on carbohydrate intake and nutrient adequacy, bridging the gap between metabolic control and nutritional optimization. Ultimately, the integration of glycemic control into AI-driven nutritional support systems is essential for achieving truly personalized metabolic management, rather than serving as a mere adjunct to DFU care.

#### 3.2.3 Summary of practical recommendations

Integrating glycemic control into DFU care pathways requires establishing individualized glycemic targets based on patient comorbidities, ulcer severity, nutritional status, and hypoglycemia risk. Clinicians should utilize CGM devices for real-time monitoring in patients with active DFUs, particularly those with an HbA1c of greater than 8.5% or evidence of GV, to enable timely dietary and therapeutic adjustments. Engaging registered dietitians or certified diabetes educators is necessary to deliver personalized medical nutrition therapy focused on carbohydrate consistency, protein adequacy, and nutrient density. Furthermore, pharmacologic therapy must be optimized with careful consideration of wound healing, cardiovascular risk, and renal function. Glycemic data should be integrated into multidisciplinary DFU care plans to ensure that metabolic targets are tracked alongside wound status, offloading adherence, and infection control. Finally, adopting emerging AI-enabled platforms that leverage CGM and dietary data can provide real-time, personalized nutritional guidance tailored to each patient’s metabolic response and healing needs.

### 3.3 Other preventive measures

In addition to nutritional support and glycemic control, several evidence-based elements of care—including regular foot inspection, therapeutic footwear, foot hygiene, patient education, podiatrist visits, custom orthotics, and the avoidance of high-risk activities—are integral to DFU prevention (46, 47). The core principles of DFU prevention require identifying the at-risk foot through comprehensive screening and determining the screening frequency based on the IWGDF risk stratification system (47). Structured, repeated, and culturally appropriate education should be provided to patients to enhance their self-care knowledge, protective behaviors, and treatment adherence. For patients with foot deformities or at high risk, custom insoles, extra-depth shoes, and other therapeutic footwear that accommodate foot biomechanics should be used to reduce the incidence of ulcers (46, 48). These preventive measures must be implemented within a multidisciplinary team (MDT) framework, as such comprehensive management strategies can significantly reduce the rate of diabetes-related lower extremity amputations. Ultimately, achieving lower barefoot plantar pressure and increasing footwear adherence are directly associated with a lower ulcer risk and shorter healing times. Emerging smart wearable technologies offer significant opportunities for the personalized prevention and management of diabetic foot complications (49). These technologies encompass smart insoles and socks equipped with multimodal sensors capable of measuring plantar pressure, shear stress, temperature, humidity, pH, and microcirculation. They also include smart insole systems featuring real-time feedback functions, AI-based automatic detection of foot ulcers, wound healing assessment tools, dynamic plantar pressure prediction models, and telemedicine platforms that enable remote data transmission and clinical intervention. However, these technologies still require rigorous validation through laboratory and clinical trials to demonstrate their accuracy, reliability, patient adherence, and effectiveness. Alongside these diagnostic wearables, active intervention modalities such as vibrotactile stimulation have shown potential in alleviating symptoms of DPN, improving balance, and enhancing localized blood flow.

## 4 DFUs and AI

AI has been applied to DFU management primarily through two paradigms, namely, image-based wound assessment and clinical risk prediction. These applications, while not nutrition-focused, establish a computational foundation—particularly deep learning architectures and multimodal data integration—that can be repurposed for nutritional and metabolic applications in DFU care.

CNNs are widely used to classify DFU images. The Smart Diabetic Foot Ulcer Scoring System (ScoreDFUNet), for example, classifies images into ulcer, infection, normal, and gangrene categories with a reported accuracy of 95.34% (3). Other studies have employed deep learning for wound segmentation (Fast R-CNN) and thermographic temperature pattern analysis for the prediction of ulcer risk (4, 50, 51). This demonstrates that CNNs can extract clinically meaningful features from photographs, a capability that is directly transferable to food image recognition for dietary assessment.

For clinical risk prediction models, machine learning algorithms, including artificial neural networks (ANNs), random forests, and decision trees, are trained on clinical and demographic data to predict DFU incidence, amputation risk, and healing outcomes (52–54). While these models currently rely on conventional variables such as age, comorbidities, wound characteristics, and routine laboratory tests, the underlying framework of multivariate time-series analysis and multimodal data fusion directly allows for the integration of CGM data, dietary logs, and nutritional biomarkers.

**Figure 3:**
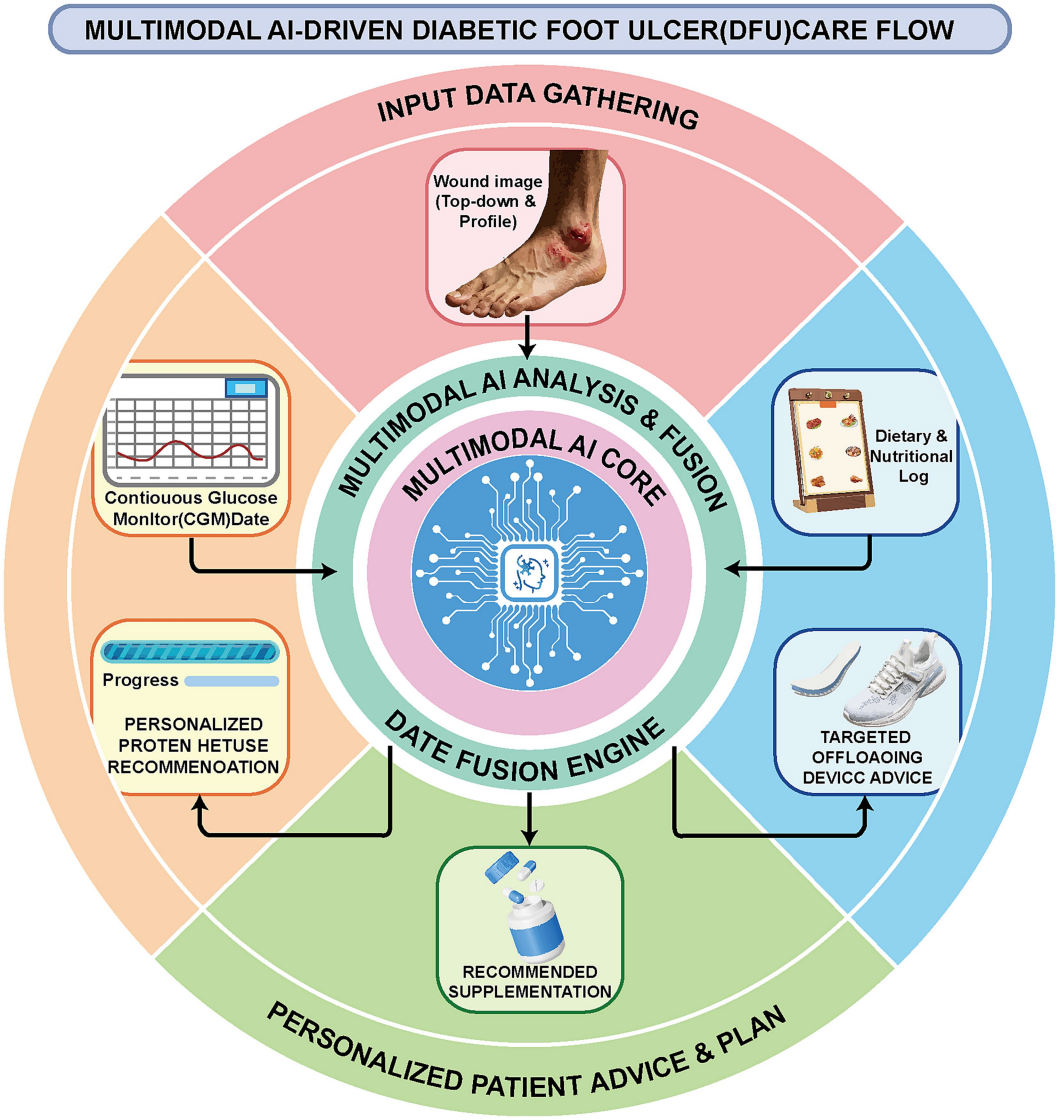
The application of artificial intelligence in diabetic foot ulcers (DFUs).

Regarding the transition to AI-enabled precision nutrition, this process relies on computational frameworks that are not inherently tied to wound images or amputation risk. CNN-based feature extraction, risk stratification *via* supervised learning, and the management of heterogeneous data streams represent foundational architectural principles that directly apply to nutritional assessment across various pathologies, offering a clear pathway for translation to DFU-specific personalized nutrition. However, these techniques can only be repurposed in principle; the actual translation is hampered not by a shortage of applicable AI techniques, but rather by a lack of DFU-validated datasets and longitudinal intervention studies that combine nutritional parameters with AI-driven decision support (Figure 3).

## 5 The application of AI in the early diagnosis and management of DFUs

**Figure 4:**
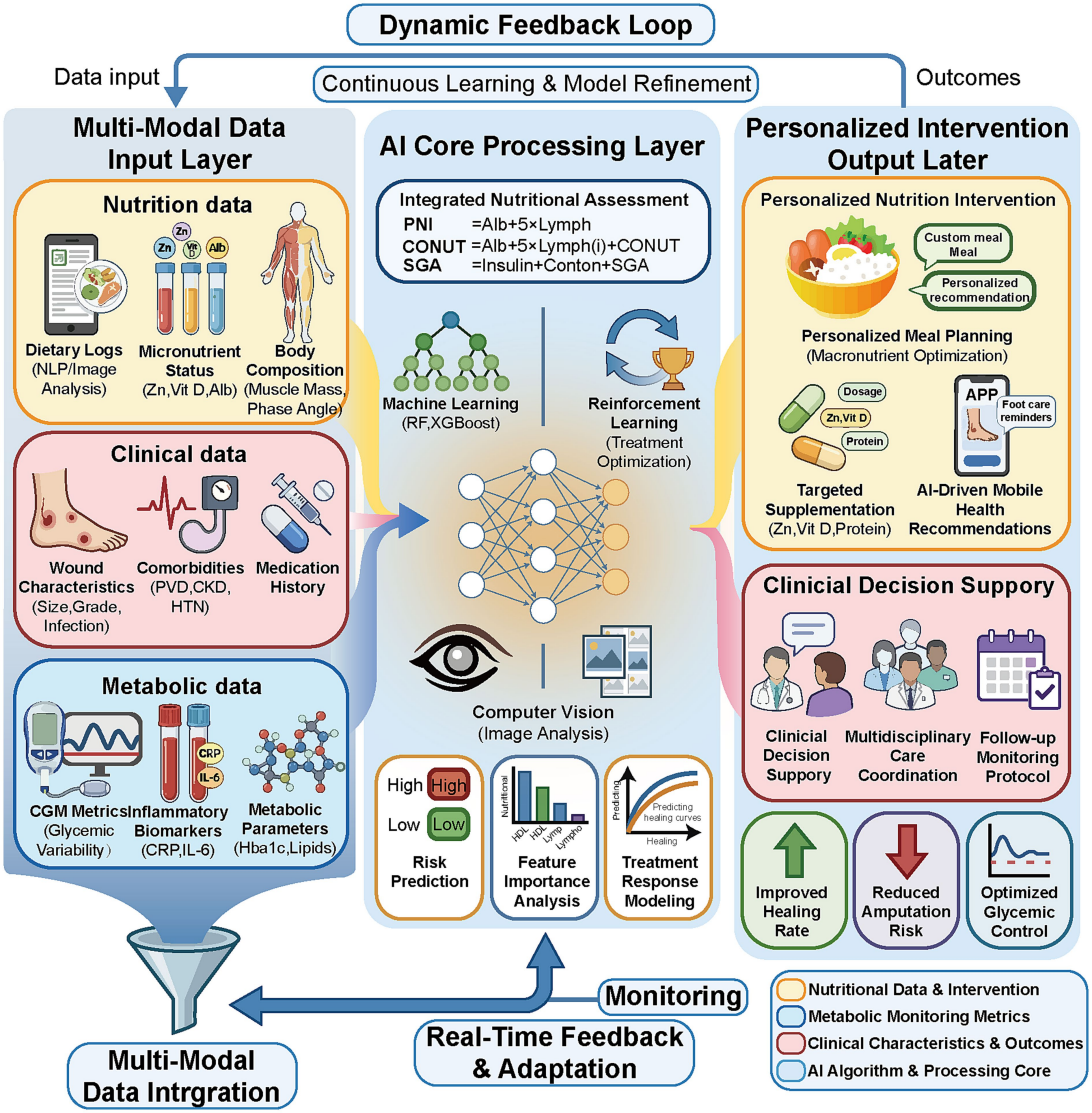
Artificial intelligence (AI)-nutrition integration framework in diabetic foot ulcer (DFU) management.

Adapting image recognition, time-series analysis, and multimodal data fusion for nutritional and metabolic purposes offers a clear pathway toward a conceptual framework for an AI-driven personalized nutrition system tailored to DFU care. Here, we critically evaluate the existing evidence across three categories: DFU-specific nutritional association studies, DFU-specific AI tools not focused on nutrition, and transferable AI-nutrition methods from other diseases. We then propose a conceptual framework for an AI-driven personalized nutrition system tailored to DFU, which directly integrates wound healing data, CGM, and dietary intake monitoring, building explicitly on foundational technical principles. The AI field is advancing rapidly, and this technology can directly support the early diagnosis and management of DFUs, playing a significant role in reducing the overall burden of this condition (Figure 4).

### 5.1 Predictive modeling

Machine learning algorithms have been applied for the prediction of DFU risk. One study developed ANNs and decision tree algorithms to predict DFU development using clinical and imaging data, achieving a 97% accuracy rate, thereby supporting early clinical intervention to reduce the amputation risk (4). Additionally, Stefanopoulos et al. developed an algorithm to predict DFU risk using ICD-9-CM and AHRQ comorbidity codes, achieving an accuracy of 79.8% (55). To estimate the risk of in-hospital amputation, Xie et al. developed an explainable prediction model that accurately predicts amputation rates in patients with DFUs (53). Ultimately, machine learning-based DFU risk prediction models that use clinical and biochemical parameters can help address the global health burden of DFUs, given the lack of reliable, non-invasive prediction tools. In a recent study, Nanda et al. built a model for DFU risk prediction using clinical and biochemical parameters derived from features learned by machine learning algorithms, which were trained and validated on a dataset obtained from diabetic individuals with and without foot ulcers (56). Wang et al. utilized a machine learning-based synthetic minority over-sampling technique to create a predictive model for minor amputation, which demonstrated high predictive validity for minor amputation requirements in newly diagnosed DFUs (54). Furthermore, Wu et al. employed machine learning to establish models for predicting the diagnosis of peripheral neuropathy and lower extremity arterial disease in diabetic patients, with the resulting models demonstrating high specificity and accuracy (57). Overall, the integration of machine learning for DFU risk prediction encompasses incidence prediction, amputation risk estimation, risk factor identification, and early warning models. Consequently, the interpretation accuracy and explainability of these machine learning models remain critical for improving the clinical management and care of patients with DFUs.

### 5.2 AI for nutritional and metabolic assessment in DFU

To the best of our knowledge, no study to date has established an AI-driven personalized nutrition intervention specifically for patients with DFU. The available evidence can be organized into three categories based on their direct relevance to AI-enabled precision nutrition for DFU, as detailed below.

#### 5.2.1 Category a: direct DFU-specific evidence

Category A includes studies that have used nutritional biomarkers—such as the PNI, CONUT score, serum zinc, albumin, and vitamin D—to predict DFU outcomes, including wound healing, amputation risk, and mortality. Representative examples include the retrospective cohort studies by Oda et al., Coşkun et al., and Nakamura et al. (7, 9, 10), all of which demonstrated that malnutrition and specific micronutrient deficiencies are independently associated with poorer DFU prognosis. However, three distinct characteristics define this body of evidence. First, these are prognostic association studies rather than AI-driven interventions, relying on conventional regression modeling without machine-learning-guided dietary recommendations, real-time nutritional feedback, or adaptive intervention. Second, the evidence remains sparse given both the small number of studies and sample sizes, and the PNI and CONUT scores are derived from routine laboratory parameters—useful for risk stratification but not directly translatable to dynamic dietary guidance. Third, causality cannot be established from retrospective designs, and it remains unclear whether abnormal nutritional parameters are drivers of poor DFU outcomes or merely markers of underlying disease severity and systemic inflammation. Importantly, a systematic review and meta-analysis of 23 studies in DFU populations by Donnelly et al. concluded that the certainty of evidence supporting nutrient supplementation for patients with DFU is very low according to GRADE assessment, featuring substantial heterogeneity and low methodological quality (18). Therefore, while the association between nutritional status and DFU outcomes is well established, direct evidence that modifying these parameters through AI-guided intervention improves DFU healing remains absent. It is important to emphasize that Category A studies are prognostic association studies using conventional regression; they do not involve AI-driven dietary recommendations, real-time feedback, or adaptive interventions.

#### 5.2.2 Category B: direct AI applications for DFU management (non-nutritional)

Category B encompasses AI tools developed specifically for DFU wound assessment (58). The most prominent example is ScoreDFUNet, a CNN-based system that classifies DFU images into ulcer, infection, normal, and gangrene categories with a reported accuracy of 95.34%, alongside high precision, recall, and F1 scores (3).

While such studies demonstrate that machine learning models can achieve exceptional technical performance metrics in DFU populations, their relevance to AI-enabled precision nutrition is indirect for two reasons. First, these tools are entirely non-nutritional; they classify wound severity, detect infection or ischemia, and predict healing trajectories based on visual wound characteristics rather than dietary intake, metabolic status, or nutritional biomarkers. Second, transferability to nutritional applications remains conceptual rather than empirical. The technical frameworks employed—including CNNs, computer vision pipelines, and multi-modal data integration—could theoretically be adapted for nutritional assessment. However, no study has validated such an adaptation in patients with DFU, leaving a distinct gap between technical feasibility and clinical utility. Although these tools demonstrate high technical performance, they are entirely non-nutritional. Their relevance to AI-enabled precision nutrition remains conceptual, as no study has validated an adaptation of such frameworks for dietary assessment in DFU populations.

#### 5.2.3 Category C: transferable AI-nutrition methods from adjacent diseases and populations

Category C includes AI-powered nutritional assessment and recommendation systems developed for non-DFU populations. These systems utilize data from cohorts involving T2DM, hemodialysis, obesity, prediabetes, and healthy adults. The methodologies represented in this category are diverse, ranging from imaging-based dietary assessment to continuous CGM-integrated glucose prediction, GPT-generated dietary recommendations, and digital twin platforms (59–63). These studies provide valuable methodological templates that are potentially applicable to DFU care. None of the Category C methods has been validated in DFU-specific cohorts. Whether their performance generalizes to patients with DFU—who have distinct metabolic demands, wound healing kinetics, and comorbidity profiles—remains unknown.

#### 5.2.4 Absence of DFU-specific validation

None of the Category C studies has been replicated in DFU cohorts. Patients with DFU have metabolic demands—including high protein and micronutrient requirements for wound healing, frequent renal impairment, polypharmacy, and a high prevalence of sarcopenia—that differ substantially from those of the populations in which these AI tools were developed. Whether the algorithmic performance observed in T2DM or hemodialysis populations is generalizable to patients with DFU remains unknown. A systematic review of AI applications for personalized dietary recommendations, which analyzed 11 studies (five RCTs, five pre-post designs, and one cross-sectional study), indicated that AI-generated interventions improved glycemic control, metabolic health, and psychological well-being in conditions such as diabetes and irritable bowel syndrome (64). However, none of the evaluated studies included patients with DFU.

#### 5.2.5 Limited external validity and scarce real-world clinical validation

A 2025 systematic review by Cofre et al. examined the validity and accuracy of AI-based dietary intake assessment methods, revealing that 61.5% of the evaluated studies were conducted in preclinical settings, with 58.3% of these exhibiting a moderate risk of bias, driven primarily by confounding factors (65). Although six studies reported correlation coefficients exceeding 0.7 between AI and traditional methods for both calorie and macronutrient estimation, these results were largely obtained under controlled laboratory conditions rather than real-world settings. Similarly, a scoping review by Sosa-Holwerda et al. concluded that the application of AI in nutrition remains in a developmental stage, focusing predominantly on dietary assessment and significantly less on malnutrition prediction, lifestyle interventions, or the mechanisms underlying diet-related pathogenesis (66). The review also highlighted that pronounced heterogeneity across studies limited the ability to draw disease-specific conclusions, and that unresolved ethical concerns—including algorithmic bias and the risk of exacerbating health disparities in specific populations—remain major challenges.

Moreover, a 2026 validation study assessed the validity of the SNAQ app, an AI-powered image-based dietary assessment tool, against doubly labeled water—the gold-standard reference for total daily energy expenditure—in females with obesity under free-living conditions. The application systematically underestimated energy intake and demonstrated an absence of individual-level agreement, yielding an intraclass correlation coefficient (ICC) of 0.00 (67). This finding is critical to DFU care, where precise nutrient estimation is vital for wound healing, and underscores the substantial performance gap between algorithmic performance under laboratory conditions and clinical viability. Separately, a comprehensive review of AI in nutrition and dietetics further noted that despite the potential for optimizing dietary tracking and generating personalized recommendations, challenges persist regarding model transparency, data ethics, limited population generalizability, and the systemic exclusion of low-resource clinical settings (68).

#### 5.2.6 Concerns regarding methodological and reporting quality

A review addressing the application of machine learning in nutritional research observed that widespread enthusiasm for these techniques frequently leads to adoption without adequate procedural understanding, resulting in non-robust study designs and unreliable outcomes (69). Key areas requiring rigorous improvement include the generation of high-quality datasets, the deployment of stringent validation techniques, the quantification of outcome stability, and the enforcement of transparent methodological reporting. These deficiencies apply directly to the Category C literature, which is characterized by small sample sizes, inconsistent validation protocols, and inflated performance claims. A 2025 systematic review analyzing 13 studies on generative AI in nutritional sciences found that while chatbots demonstrate utility for rudimentary dietary guidance, they fail to adequately manage complex clinical phenotypes and remain susceptible to generating spurious clinical output (70). The review highlighted pronounced methodological heterogeneity, a reliance on subjective or poorly operationalized metrics, and the absence of reproducibility testing, concluding that AI chatbots are currently unviable for unsupervised clinical use.

#### 5.2.7 Heterogeneity in AI-nutrition research restricts disease-specific conclusion

The broader field of AI-driven nutritional research remains at an early, highly heterogeneous stage. A systematic review of dynamic nutrient profiling for personalized diet planning found that while advanced algorithmic approaches integrating real-time nutritional assessment with individualized recommendations are emerging, methodological quality remains variable, and large-scale validation within specific patient populations is lacking (71). Similarly, a comprehensive review of AI-driven personalized nutrition applications concluded that although advanced methodologies—including deep learning, federated learning, and computer vision—are transforming static dietary models into dynamic frameworks, major challenges persist regarding algorithmic transparency, data privacy, and equitable access, areas that are critically underexplored in the DFU context (72).

#### 5.2.8 Synthesis of methodological gaps

Collectively, the reviewed literature exposes several interconnected methodological gaps that constrain the current evidence base for AI-enabled precision nutrition in DFU management.

No DFU-specific, AI-driven nutritional interventions have been published to date. Category C methods—while methodologically promising—originate from adjacent diseases (general type 2 diabetes, hemodialysis, prediabetes, healthy adults) and require dedicated validation in DFU populations before any clinical translation can be considered. The absence of prospective, standardized nutritional phenotyping in DFU cohorts (see section 6.2) is the primary reason this validation has not yet been performed. Whether an AI system can safely and effectively guide personalized dietary changes to improve wound healing trajectories remains an open question. The technical building blocks for such interventions have been developed and validated exclusively in non-DFU populations. While these methods show promise, their performance, safety, and acceptability in patients with DFU require dedicated investigation. Even within adjacent populations, clinical validation under free-living conditions remains limited. Most AI-based dietary assessment tools have been evaluated under controlled laboratory conditions or within small sample cohorts and show performance deficits when evaluated against gold-standard physiological methods. Finally, methodological quality and reporting standards for AI applications in nutritional science remain inconsistent. Furthermore, methodological quality and reporting standards for AI in nutrition remain inconsistent across the literature. Poor validation practices, unadjusted confounding, a lack of transparency, over-optimistic performance claims, and limited population generalizability are common concerns that must be addressed in future DFU-focused research. Consequently, while the foundational elements for AI-enabled precision nutrition in DFU management exist—supported by robust associations between nutritional biomarkers and clinical endpoints, alongside mature AI architectures for image analysis and extensive methodological work in adjacent disease areas—their application to patients with DFU remains conceptual. Table 2 organizes the available literature by evidence level and DFU-specificity, establishing a structured framework to assess the maturity of the current evidence base.

**Table 2:** Evidence levels of AI-nutrition studies relevant to DFU.

Technique	Nutritional application	Outcome/goal	Evidence level (DFU-specificity)	References
Direct DFU-specific prognostic models using nutritional biomarkers				
Multivariate Cox regression, competing risk models	PNI and CONUT scores in DFU patients (*n* = 349)	Higher PNI and lower CONUT independently predict wound healing (PNI HR = 1.22; CONUT HR = 0.80)	Direct DFU evidence prognostic models (no AI-driven dietary intervention)	(9)
Multivariate logistic regression, ROC analysis	PNI in DFU patients (*n* = 386)	PNI predicts amputation (AUC = 0.937; OR = 81.8 for PNI ≤ 39.005; sensitivity 82.7%, specificity 93.1%)	Direct DFU evidence—prognostic models (no AI-driven dietary intervention)	(7)
Multivariable logistic regression	CONUT and serum zinc in DFU patients (*n* = 319)	High CONUT independent factor for DFU severity; low zinc (<13 mmol/L) independent factor for non-healing	Direct DFU evidence—prognostic models (no AI-driven dietary intervention)	(10)
Direct AI applications for DFU management (non-nutrition)				
CNN-based Score DFUNet	DFU image classification (ulcer, infection, normal, gangrene)	Classification accuracy 95.34%; outperforms junior and mid-level dermatologists	Direct DFU AI tools—not nutrition-focused	(3)
Fast R-CNN with transfer learning	Diabetic foot wound image segmentation and object detection	Detection efficiency up to 89%; supports treatment plan execution	Direct DFU AI tools—not nutrition-focused	(50)
Transferable AI-nutrition methods from adjacent diseases/populations				
GPT-based dietary recommendation system	Hemodialysis patients (*n* = 88)	GPT-based counseling reduced pre-dialysis serum potassium vs. conventional education (4.57 vs. 4.84 mmol/L, *p* = 0.004); hyperkalemia prevalence 39.8% → 25.0% (*p* = 0.036)	Transferable from adjacent field—not validated in DFU	(62)
XGBoost, SARIMA, Prophet + CGM	General type 2 diabetes management	AI-enhanced time series analysis for personalized dietary intervention based on CGM data; XGBoost best predictive performance	Transferable from adjacent field—not validated in DFU	(60)
ChatGPT evaluation	Type 2 diabetes and metabolic syndrome	ChatGPT-generated nutritional guidance showed good-excellent clarity but significant gaps in dietary recommendations and micronutrient adequacy	Transferable from adjacent field—not validated in DFU	(61)
ChatGPT evaluation	Type 2 diabetes (meal planning)	ChatGPT generated generally accurate advice aligned with ADA guidelines but menus tended toward low-carb patterns; fabricated non-existent references	Transferable from adjacent field—not validated in DFU	(135)
ChatGPT (perspective)	Personalized obesity treatment	ChatGPT can provide personalized nutrition plans, exercise programs, and psychological support; limitations include lack of emotional intelligence and accountability	Transferable from adjacent field—not validated in DFU	(136)
Image recognition (Keenoa app)	Adults including those with diabetes (*n* = 136)	Moderate-to-strong validity against ASA24 for energy, macronutrients, micronutrients; superior usability (SUS 77 vs. 53)	Transferable from adjacent field—not validated in DFU	(59)
Machine learning (PPGT algorithm)	Newly diagnosed T2DM (crossover RCT)	PPT diet significantly improved CGM-based glycemic measures vs. Mediterranean diet; 6-month intervention improved HbA1c, fasting glucose, HOMA-IR; 61% achieved diabetes remission	Transferable from adjacent field—not validated in DFU	(137)
Digital twin-enabled personalized nutrition	Type 2 diabetes with MAFLD (1-year RCT)	DT-enabled personalized nutrition improved HbA1c (−2.9% vs. − 0.3%); 72.7% T2D remission; reduced MRI-derived liver fat (5.5% vs. 10.9%)	Transferable from adjacent field—not validated in DFU	(63)
AI-discovered functional ingredient (NRT_N0G5IJ)	Prediabetic adults (12-week trial)	Daily pea protein hydrolysate supplementation reduced HbA1c by 0.12% (*p* = 0.013); enhanced glucose uptake *in vitro*	Transferable from adjacent field—not validated in DFU	(138)
Deep learning/supervised learning (review)	Automatic diet monitoring and personalized diet recommendations for diabetes	Food recognition accuracy >85%; personalized ML-based diets lower post-meal blood glucose	Transferable from adjacent field—not validated in DFU	(139)
Image recognition + nutrient calculation (review)	General diabetes dietary management	Automated nutrition monitoring and carbohydrate counting; comparable accuracy to dietitian assessments	Transferable from adjacent field—not validated in DFU	(140)
AI4FoodDB database	Public database for e-Health nutrition and lifestyle (weight loss intervention)	Centralizes food images, wearable sensor data, validated questionnaires, and biological samples from 100 individuals	Transferable from adjacent field—not validated in DFU	(141)

### 5.3 Integrating CGM and body composition with AI for DFU management

The technical feasibility of such an integrated system relies on computational architectures previously validated on DFU clinical datasets generated for alternative diagnostic purposes, as detailed in Section 4. Specifically, the CNN architectures that achieve high accuracy in DFU wound classification—such as ScoreDFUNet—can be retrained on food image databases to recognize meal components and estimate portion sizes. Similarly, risk prediction models that currently integrate clinical and laboratory metrics to forecast amputation can incorporate CGM-derived GV and serial nutritional biomarker measurements. Thus, the divide between existing DFU-AI applications and nutritional informatics is methodological rather than technical; the underlying architectures exist but require training and testing on DFU-specific nutritional outcomes.

The following framework is presented as a conceptual model to guide future research and development rather than a declaration of clinical readiness. While direct clinical evidence is currently unavailable, it is possible to define how an AI-driven nutritional intervention system would operate in the DFU care pathway, as detailed below. This framework serves as a conceptual model to guide future research and development rather than a declaration of clinical readiness.

A DFU-specific AI nutrition tool would integrate four core modules: a dietary intake sensor that uses a smartphone-based image recognition system—which must be validated for wound-healing populations—to estimate protein, energy, and micronutrient consumption from meal photos; a metabolic monitor that processes CGM data alongside optional inputs from indirect calorimetry or wearable physical activity sensors (73); a clinical data integrator that extracts electronic health record data regarding wound characteristics (area, depth, infection, ischemia), nutritional biomarkers (albumin, PNI, CONUT, zinc, vitamin D), and medication lists; and a personalized recommendation engine driven by reinforcement learning that models individual glycemic and healing responses to specific dietary inputs to generate just-in-time behavioral interventions.

Consider a 62-year-old male patient with a chronic neuropathic DFU of 8 weeks’ duration, a baseline HbA1c of 8.9% (74 mmol/mol), serum albumin of 3.2 g/dL, and a CONUT score of 5, indicating that moderate malnutrition is present (11). The patient is monitored *via* a CGM sensor and records dietary intake using a validated food photography application for 1 week. The AI model determines that the patient’s time-in-range (70–180 mg/dL) is limited to 45%, postprandial lunch glucose excursions exceed 250 mg/dL, and midday protein intake averages 15 g. Based on these parameters, the recommendation engine could generate a personalized intervention that redistributes the carbohydrate load by delivering a time-specific prompt for a lower-glycemic lunch option. To optimize protein intake, the system instructs the patient to consume a 20 g whey protein supplement prior to the afternoon CGM-predicted glucose trough and schedules oral zinc and vitamin D supplementation, based on documented laboratory deficiencies. After 2 weeks, the system could evaluate wound area through automated wound segmentation of smartphone images alongside updated CGM metrics. If the healing velocity remains below the clinical target, the model could adjust the intervention by recommending a leucine-enriched protein formulation or a restricted evening eating window to enhance overnight glycemic stability.

#### 5.3.1 Key differentiators from generic AI nutrition apps

Unlike general-population tools, a DFU-specific AI system must account for wound healing kinetics, the catabolic state of chronic inflammation, and frequent renal or hepatic comorbidities that alter nutrient metabolism. The system must also integrate directly into multidisciplinary DFU care pathways, alerting the attending podiatrist or dietitian when wound healing stalls despite apparent dietary adherence. Furthermore, the model must be trained and validated on DFU-specific ground-truth outcomes rather than intermediate metrics such as weight loss or HbA1c reduction. Consequently, future prospective studies must randomize patients to AI-guided interventions *versus* standard nutrition counseling, and evaluate healing time, amputation-free survival, and quality of life as primary endpoints.

#### 5.3.2 Validation roadmap

Before clinical adoption, this conceptual framework requires a stepwise validation process. The initial phase must establish technical feasibility, determining whether a CNN-based food image classifier can achieve acceptable accuracy when deployed by DFU patients managing typical comorbidities and polypharmacy. Following technical validation, a pilot clinical study utilizing a single-arm, pre-post design in 30 to 50 DFU patients is required to evaluate safety, user acceptability, and preliminary signals of improved healing. The final phase demands a multi-center RCT comparing AI-guided personalized nutrition to standard care, powered specifically to evaluate healing rates and the incidence of amputation. To date, none of these translational steps has been executed.

#### 5.3.3 AI models leveraging CGM data for DFU healing prediction and glycemic management

The integration of CGM with AI provides new approaches for predicting DFU healing trajectories and personalizing metabolic management. Traditional glycemic metrics, such as HbA1c, provide only a static snapshot of glycemic control, whereas CGM-derived dynamic metrics—including TIR, GV, and the GMI—offer real-time, high-resolution data that machine learning models can utilize to forecast wound healing outcomes. A prospective study by Ortiz-Zúñiga et al. demonstrated that there was a significant inverse correlation between TIR and DFU healing time, indicating that higher TIR is associated with accelerated ulcer closure in uncomplicated DFUs (25). These findings indicate that beyond functioning as markers of systemic glycemic control, CGM-derived metrics serve as dynamic indicators of tissue repair trajectories.

Emerging AI architectures can transform raw CGM data into actionable clinical predictions. Machine learning models, including random forests and gradient boosting algorithms, have been trained on CGM time-series data to predict GV and identify high-risk glycemic patterns in cohorts with diabetes. More recently, the paradigm of “CGM Data Analysis 2.0” has been proposed, which employs functional data analysis and AI to provide a detailed understanding of glucose fluctuations and support personalized diabetes management strategies (74). However, the direct application of these AI-CGM models to DFU-specific outcomes—such as predicting delayed healing, infection, or amputation risk—remains unvalidated. While technical feasibility has been established, no study to date has developed or externally validated an AI model that uses CGM data specifically to predict DFU healing trajectories in a prospective cohort. This evidence gap necessitates the development and validation of CGM-integrated AI models tailored to the specific metabolic and wound healing dynamics of patients with DFUs.

### 5.4 Image analysis

Studies have explored the use of AI-driven image recognition technologies, such as CNNs, to predict the risk of DFU development. Das et al. proposed a method combining handcrafted and deep CNN features for effective DFU diagnosis to assist clinicians in decision-making (75). Alavi and Akhoundi developed a deep subspace analysis pipeline for semi-supervised multi-label classification of DFUs using random forests, which demonstrated a significant improvement compared to classical methods (76). Xu et al. developed a class knowledge bank (CKB) framework for DFU image classification, designed to capture and encode class-specific features of infection and ischemia; this approach enhanced classification performance while remaining computationally efficient (77). To improve specialized imaging modalities, Bouallal et al. proposed a Double Encoder-ResUnet architecture for diabetic foot thermal image segmentation (78). For automated detection, Huang et al. utilized a Fast R-CNN method to identify diabetic foot wounds, achieving a detection efficiency of 90% (50). Sathya Preiya and Kumar employed a deep recurrent neural network (DRNN) to extract features from foot images, demonstrating 99.32% accuracy for feature extraction and DFU classification (4). Similarly, Khosa et al. developed a CNN-based model for automatic DFU recognition using multi-level thermographic imaging data, which outperformed alternative models in diagnostic accuracy (51). To address clinical interpretability, Biswas et al. introduced XAI-FusionNet, which bases DFU detection on multi-scale feature fusion combined with explainable AI (79). These studies demonstrate that AI-driven image recognition technologies can accurately classify diseases and assess patient risk profiles.

### 5.5 Thermography and temperature monitoring

Foot temperature variations can indicate early ulcer onset. AI can identify abnormal thermal patterns associated with DFUs through thermographic imaging, enabling proactive clinical management, cost-effective interventions, and rapid monitoring of pedal health.

In a clinical evaluation, Isaac et al. investigated the impact of a DFU prevention program incorporating foot temperature monitoring on hospitalizations, emergency department visits, and recurrence rates, and found that the program decreased healthcare resource utilization among patients at high risk for DFUs (80). To quantify regional temperature variations, Ghosh et al. monitored skin temperatures on affected and unaffected feet using infrared dermal thermometry and established that a temperature gradient of ≥1 °C could predict impaired DFU healing (81). Transitioning this technology to patient-led care, Oe et al. assessed the effects of an infrared thermography smartphone attachment and noted that this self-monitoring device promotes patient engagement in preventative self-care behaviors that help prevent DFU development (82). From an economic perspective, Brooks et al. compared the cost-effectiveness of remote foot temperature monitoring combined with standard of care *versus* standard of care alone; the results demonstrated that the combination significantly reduced healthcare costs for patients with diabetic neuropathy at moderate-to-high risk of ulceration (83). Supporting these findings, a meta-analysis conducted by Golledge et al. examined the efficacy of at-home foot temperature monitoring programs and showed that patients allocated to these protocols experienced a reduced risk of DFU development (84). Additionally, in a RCT evaluating a thermography-driven preventive foot care protocol, Qin et al. found that the intervention group achieved a significant reduction in DFU recurrence (85). Finally, a systematic review by Thakku Sivakumar et al. indicated that while technical and clinical implementation challenges remain, thermography shows promise as a predictive tool for DFU risk (86). Collectively, these data demonstrate that AI-compatible thermography and temperature monitoring protocols can accurately predict and prevent DFU development within clinical and residential settings.

### 5.6 Automated scoring systems

Automated scoring systems with enhanced consistency and reliability—such as ScoreDFUNet, which integrates AI and image analysis for the evaluation of DFU severity—can assist healthcare professionals in developing individualized treatment strategies by quantifying ulcer characteristics, including size and infection status, and providing predictive diagnostic scores (3).

To guide clinical selection among various validation tools, Monteiro-Soares et al. assessed 37 published DFU classification systems, providing evidence-based recommendations on using specific classifications for distinct clinical purposes (87, 88). Predictive scoring systems also help identify patients at risk for severe complications. Reddy et al. developed a novel risk score system identifying age, diabetes duration, amputation history, ulcer depth, ankle-brachial index, infection severity, peripheral neuropathy, peripheral arterial disease, and wound-based parameters as significant predictors of lower extremity amputation in patients with DFUs (89). Similarly, established scoring frameworks like the SINBAD or Wagner-Armstrong scales provide useful prognostic indicators for amputation risk in patients with DFUs, independent of patient age (90). Beyond wound-specific parameters, logistic regression models designed to predict DFU incidence in patients with diabetes have identified high-density lipoprotein (HDL) cholesterol as a negative predictor of ulcer development (91). Furthermore, utilizing the Wagner and IWGDF grading scales, researchers have highlighted the significance of the neutrophil-to-lymphocyte ratio (NLR) and platelet-to-lymphocyte ratio (PLR) as systemic markers in DFUs, serving as potential therapeutic targets (92). In a systematic review of prognostic tools, researchers identified four accurate DFU prediction models, with the PODUS 2020 model achieving the highest prognostic accuracy (93). Additionally, a comparative analysis of the newly revised DIRECT coding system against five existing classification systems for lower extremity amputation prediction demonstrated that the former was highly effective for clinical prediction and treatment selection (94). Collectively, these studies demonstrate that integrating automated scoring systems and validated risk frameworks improves clinical decision-making, refines risk stratification, and supports targeted therapeutic interventions for patients with diabetes.

### 5.7 Telehealth and remote monitoring

AI-driven telehealth applications and remote foot health monitoring support patient self-management by allowing individuals to track their foot conditions and receive timely feedback from healthcare providers. This approach expands clinical touchpoints for patients with limited access to brick-and-mortar healthcare facilities, improving overall patient autonomy and care efficiency (4).

To identify early signs of tissue stress, Banks et al. evaluated a remote temperature monitoring mat for the early detection of diabetic foot complications, emphasizing its role in prompting timely clinical intervention (95). Dmitriyeva et al. demonstrated the utility of remote monitoring and treatment frameworks for DFUs during the COVID-19 pandemic, providing an objective algorithm for determining the ulcer infection risk (96). Synthesizing broader digital trends, Najafi and Mishra conducted a narrative review of digital health technologies for patients with diabetes, advocating for a paradigm shift toward community-based prevention in the management of DFUs (97). Furthermore, Kong et al. presented a case report detailing a patient with diabetes whose DFUs were successfully managed *via* a remote, patient-facing wound care smartphone application, illustrating the clinical effectiveness of remote wound assessment (98). However, Littman et al. assessed the effectiveness of remote foot temperature monitoring, finding no reduction in the risk of lower extremity amputation associated with that system (99). These studies on telehealth and remote monitoring for predicting the risk of DFUs highlight the importance of the use of innovative technologies for their early detection and prevention.

### 5.8 Data mining and risk assessment

Utilizing AI-driven data mining methods to analyze large datasets from electronic patient records allows researchers to uncover hidden patterns and correlations, thereby supporting targeted risk assessment and management strategies, and ultimately improving clinical outcomes for patients with diabetes (100).

Van Netten et al. leveraged big data science, incorporating structured biomechanical and behavioral profiling to identify additional risk factors for diabetic foot disease, with their results supporting a paradigm shift toward personalized medicine (101). Peng et al. constructed a predictive nomogram designed to quantify amputation risk in patients with existing DFUs, providing an objective tool to mitigate amputation risk through early clinical identification (102). For primary prevention, Chappell et al. developed and validated a clinical prediction rule for diabetic foot ulceration, establishing a framework for reducing DFU development by redirecting specialized care resources to high-risk individuals (103). Using readily accessible clinical variables, Aan de Stegge et al. developed and validated a prediction model for foot ulcer recurrence in people with diabetes (104). Finally, Xie et al. developed an explainable machine learning model that could accurately predict in-hospital amputation rates for patients with DFUs (53).

### 5.9 Multidisciplinary approaches

AI combines insights from various healthcare disciplines to support a multidisciplinary approach to DFU management, ensuring comprehensive care and enhancing treatment effectiveness for patients with diabetes (105).

Mousa et al. designed AI-based frameworks utilizing ANN and decision tree algorithms to predict DFU development (52). These machine learning methodologies serve to predict the wound healing process, assess amputation risk, and assist in designing personalized treatment plans for individual patients. Emphasizing the clinical utility of these integrated strategies, Wang et al. argued that the application of machine learning, combined with interdisciplinary collaboration for DFU prediction, holds significant value in clinical practice and provides substantial reference data to guide medical teams (3). The evidence provided in this review demonstrates that integrating AI into early DFU diagnosis and clinical management offers high potential for improving patient outcomes. Employing predictive models, image analysis, thermography, automated ulcer scoring systems, telehealth, and data mining expands opportunities for early detection, ensures continuous monitoring, and reduces the overall DFU burden for both patients and the healthcare system.

The integration of AI into multidisciplinary care pathways for DFUs offers an innovative strategy to address this clinically challenging condition. Managing DFUs requires an interdisciplinary approach spanning multiple specialties—including endocrinology, wound care, vascular surgery, and infectious disease—to achieve optimal clinical outcomes (106). The historic synergy between the medical, engineering, and computer science communities can be advanced by leveraging modern AI methods for data analysis, computer-assisted diagnosis, and treatment planning. Appropriate AI software can synthesize multimodal data obtained from patients with ulcers, allowing for the precise prediction of ulcer activity and progression. Moreover, these automated tools can assess and forecast the risk of secondary complications, and the most effective and standard treatment for the largest possible number of patients can be determined. Ultimately, these integrated workflows aim to improve health outcomes and extend life expectancy among individuals affected by ulcers (106, 107). This systemic approach aligns with Realistic Evaluation principles, where context-specific “what works, for whom, and under what circumstances” frameworks are required (106). Furthermore, utilizing an AI platform as a centralized communication hub enables real-time data sharing across the care team, which ensures clinical alignment and avoids the fragmentation of patient care (108, 109). A pivotal advancement resides in the capacity of AI to enhance clinical workflows through predictive analytics. Machine learning algorithms trained on extensive DFU datasets can discern nuanced patterns in wound characteristics that correlate with healing trajectories, thereby enabling timely intervention (107, 110). These models can also integrate patient-reported outcomes and behavioral data to refine risk stratification (106). Using AI to automate routine tasks, such as performing wound measurement through image analysis or notifying clinicians of potential infection, serves to reallocate clinical hours to high-value interventions, including patient education and multidisciplinary care planning (107). Applying AI to patient-centered care remains a recurring theme in the healthcare literature. Several studies indicate that applying AI tools can empower patients to manage their condition by improving health literacy and increasing engagement in their own care. For instance, some interactive AI frameworks incorporate specialized communication strategies, such as the conversational use of humor, to build stronger relationships between healthcare providers and patients (106). The effective integration of AI into DFU management necessitates the resolution of several fundamental challenges. These include data quality and interoperability, involving the harmonization of heterogeneous datasets derived from primary and secondary care settings, which is essential for the development of robust, generalizable models (106, 111); and ethical considerations, comprising the mitigation of algorithmic bias and disparities in technology access, thereby ensuring equitable care delivery across diverse patient populations (110, 111). Ultimately, AI-enabled multidisciplinary pathways hold promise for reducing the global burden of DFUs, but their long-term clinical impact will depend on rigorous validation, broad stakeholder engagement, and seamless integration into existing healthcare ecosystems (110, 111).

## 6 Challenges associated with AI application

### 6.1 General barriers to AI implementation in DFU care

Several challenges impede the clinical integration and operational effectiveness of AI in DFU management. A primary obstacle stems from the lack of objectivity and consistency in conventional DFU assessment methods, which significantly increases amputation risks and highlights the urgent need for standardized diagnostic tools (3). To address this, Wang et al. evaluated ScoreDFUNet, a deep learning-based model, which demonstrated classification performance exceeding that of junior and mid-level clinicians (3). However, widespread clinical adoption requires extensive validation from expert clinicians to verify the long-term reliability and stability of such frameworks. Integrating these technologies into existing clinical workflows presents another significant barrier. Many healthcare providers resist adopting unfamiliar digital tools due to concerns regarding operational complexity, the demand for specialized training, and the potential disruption of established practices. Accommodating AI systems often necessitates substantial modifications to clinical protocols, which may prove unfeasible in resource-constrained environments and further exacerbate institutional resistance (3). Furthermore, the predictive accuracy of machine learning models heavily relies on the quality and volume of the underlying training data. Incomplete, biased, or unrepresentative datasets can cause algorithms to generate inaccurate risk assessments across different demographic groups or clinical settings, ultimately limiting their applicability and effectiveness (52).

Patient privacy and data security introduce critical ethical implications that must be addressed when deploying AI in healthcare. The collection and analysis of sensitive clinical data raise significant concerns regarding how information is stored, shared, and used. Compliance with stringent legal frameworks, such as the Health Insurance Portability and Accountability Act (HIPAA) in the United States or the General Data Protection Regulation (GDPR) in Europe, increases the operational complexity of implementing AI solutions in clinical practice (52). Additionally, the multifactorial nature of DFUs makes designing predictive systems exceptionally difficult. To accurately forecast and manage DFU outcomes, algorithms must synthesize a wide range of overlapping variables and patient-specific physiological factors (112). Finally, the necessity of a multidisciplinary approach poses an ongoing integration challenge. An effective AI infrastructure must be designed to support collaboration among various healthcare professionals—including endocrinologists, podiatrists, and wound care specialists—rather than creating isolated silos of clinical information (113). Despite considerable promise for enhancing DFU assessment and management, AI faces substantial implementation barriers. Critical challenges—including methodological standardization, seamless integration into clinical workflows, data quality and accessibility, ethical governance, disease complexity, and the need for multidisciplinary collaboration—must be systematically addressed to ensure tangible improvements in patient outcomes.

### 6.2 Nutritional and metabolic data scarcity in current DFU cohorts

A fundamental and methodologically critical challenge concerns the systematic underrepresentation of granular nutritional and metabolic data in existing DFU cohorts. While observational studies establish the importance of nutritional status—quantified by composite indices such as the PNI or the CONUT score, alongside micronutrient biomarkers such as zinc and vitamin D—the vast majority of these parameters are derived from routine blood tests rather than from dedicated, standardized dietary assessments. The absence of prospective dietary intake records, longitudinal micronutrient profiling, body composition metrics, and CGM data from DFU datasets constitutes a significant informational deficit. This limitation fundamentally constrains the development of AI-enabled precision nutrition models, because machine learning algorithms require consistent, high-dimensional input features to learn generalizable patterns—variables that are currently not collected in a standardized manner across most DFU clinical registries.

This deficiency is not merely a technical oversight but rather reflects a deeper epistemological limitation in the current DFU research paradigm. Most existing cohort studies were designed to study wound healing, infection, or amputation, with nutritional variables evaluated as secondary covariates rather than primary exposures. Consequently, even when associations between nutritional parameters and DFU outcomes are reported, the lack of comprehensive dietary phenotyping precludes causal inference regarding whether observed deficiencies stem from inadequate intake, increased metabolic demand, malabsorption, or systemic inflammation. A recent GRADE evaluation by Donnelly et al. rated the certainty of evidence for nutrient supplementation in DFU populations as low or very low, reflecting major methodological limitations that stem directly from the poor nutritional data infrastructure in existing cohorts (18). Without prospective, standardized nutritional phenotyping, AI models will remain limited to correlational analyses of proxy biomarkers. This data deficit directly explains why even transferable AI-nutrition methods from Category C cannot be directly applied to DFU care: they were trained on populations with routine dietary intake records, repeated body composition measurements, or CGM data—variables that are not systematically collected in most existing DFU cohorts.

Empirical studies illustrate the severity of this phenotypic deficit. In a cohort of 102 patients with diabetes-related foot complications, Martin et al. reported that despite a mean body mass index (BMI) falling within the overweight range, participants seldom met recommended intakes for fruits, vegetables, or protein (114). Biochemical assessments demonstrated widespread deficiencies in vitamin D, zinc, iron, and vitamin C. Critically, the authors assessed dietary intake using a non-validated *ad hoc* questionnaire—a common yet methodologically problematic practice in current literature. These observations reveal a distinct clinical contradiction: nutritional inadequacies remain demonstrably common, yet the profound methodological heterogeneity across studies—ranging from single serum biomarkers to composite scores and unvalidated questionnaires—prevents meaningful data harmonization, creating an operational barrier to training generalizable AI models.

Furthermore, nutritional status fluctuates continuously throughout DFU care, shifting alongside disease progression, metabolic stress, medication use, and behavioral factors. A single cross-sectional measurement of serum albumin or zinc at hospital admission captures a momentary snapshot that frequently reflects acute-phase illness and systemic inflammation rather than habitual nutritional reserves. The lack of serial, repeated nutritional measurements across the wound healing continuum severely limits the training of longitudinal predictive algorithms. Addressing this data scarcity requires a clear paradigm shift: moving from *ad hoc*, cross-sectional biomarker snapshots to the prospective, protocol-driven collection of comprehensive dietary, metabolic, and body composition data using validated instruments.

### 6.3 Inherent limitations of AI-driven dietary assessment methods

Beyond the absence of standardized dietary data, the quality of AI-based nutritional assessment is further constrained by intrinsic limitations of the dietary assessment methods themselves, whether traditional or AI-powered.

#### 6.3.1 Recall bias and measurement error

Food Frequency Questionnaires (FFQs) and 24-h dietary recalls—the most frequently used tools in diabetic cohorts—rely on short- and medium-term memory and are susceptible to recall bias and researcher bias. In DFU populations, chronic wound-related distress, polypharmacy, and cognitive burden may further compromise self-report accuracy. These issues are not solved by AI. Even AI-powered image-based tools, while showing promise in controlled laboratory settings, have demonstrated substantial performance gaps under real-world conditions. A validation study against doubly labeled water—the physiological gold standard for energy expenditure—in 20 females with obesity reported that the SNAQ app systematically underestimated energy intake by 25% (bias −817 kcal/day), with 95% limits of agreement ranging from −3,707 to +2073 kcal/day and negligible individual-level reliability (ICC = 0.00). This finding underscores the challenge associated with translating algorithmic performance in preclinical settings into clinical feasibility in target populations, and further highlights that current AI-powered dietary assessment methods remain unsuitable for unsupervised use in individuals with metabolic disorders such as obesity or DUCs (67). A systematic review of AI-based dietary intake assessment methods found that 61.5% of included studies were conducted in preclinical settings, and 61.5% had a moderate risk of bias, with confounding bias being the most common (65). Critically, these estimates derive from studies in general adult populations, not DFU-specific cohorts. Notably, the magnitude of recall bias in DFU patients remains unexplored.

#### 6.3.2 Lack of a ground truth for dietary intake

Unlike laboratory-based biomarkers that can be measured against reference standards, habitual dietary intake lacks a universally accepted “ground truth” for validation in free-living populations. Doubly labeled water is accurate for total energy expenditure but does not provide macronutrient or food-specific information. Weighed food records are labor-intensive and subject to reactivity. Consequently, performance metrics reported for AI dietary assessment tools—such as food recognition accuracy and calorie estimation error—are relative to a chosen reference method, each with its own biases. This absence of a practical, scalable gold standard means that claims of “high accuracy” must be interpreted cautiously.

#### 6.3.3 Privacy and data security of meal photographs

The use of meal photographs introduces unique privacy concerns. Unlike de-identified clinical laboratory data, meal images may inadvertently capture identifiable background information—including family members, home environments, or medication packaging—and reveal sensitive details about a patient’s eating habits, socioeconomic status, or living conditions. Storage, transmission, and processing of such images create risks of data breaches and unauthorized re-identification, particularly when images are uploaded to cloud-based AI servers. Compliance with data protection regulations, such as HIPAA and GDPR, requires explicit consent, robust encryption, and, ideally, on-device processing to minimize data exposure. However, the computational requirements for on-device image recognition may be prohibitive in low-resource settings, creating a conflict between privacy preservation and technical feasibility.

Consequently, recall bias, the absence of a practical ground truth, and privacy concerns inherent to AI-based dietary assessment must be explicitly documented and, where possible, mitigated through objective nutritional biomarkers, repeated measurements, and privacy-preserving architectures when designing AI-driven nutritional studies involving DFU populations.

### 6.4 Economic barriers in resource-limited settings

A further, often overlooked barrier to widespread clinical deployment concerns the economic and infrastructural barriers to the implementation of multimodal AI systems—including CGM devices, high-resolution cameras for meal photography, and cloud-based computational infrastructure—in low- and middle-income countries (LMICs), where the global burden of DFU is disproportionately concentrated. In these countries, the cost of a single CGM sensor is often prohibitively high—frequently exceeding the monthly income of many individuals—and such devices are rarely covered by insurance. Beyond device costs, the necessary infrastructure (reliable electricity, high-speed internet, cloud storage, and ongoing technical support) is frequently unavailable in rural or remote regions, precisely where the clinical burden of DFU is highest. Even in high-income countries, a systematic review of telehealth interventions for DFU found no consistent cost savings compared to standard care (2, 115). Consequently, the economic feasibility of deploying the AI-driven personalized nutrition systems envisioned in this review in resource-limited settings is unknown, a limitation that must be addressed in future health economics research alongside technical development.

### 6.5 Validation gap: single-center designs and lack of external validation

A systematic review of machine learning models for DFU outcomes noted that most studies were subject to a high risk of bias due to inconsistent outcome definitions, small sample sizes, and inadequate handling of missing data (116). Few models have undergone external validation or assessed calibration, limiting clinical applicability. For instance, ScoreDFUNet and the Fast R-CNN model (50) were developed on single-center datasets; although the former was tested on a challenge dataset, independent external validation in a separate clinical cohort remains absent to the best of our knowledge. Even when external validation has been attempted—such as a machine-learning model for DFU risk prediction trained on NHANES and tested on a Chinese cohort—the AUC decreased from 0.81 in the derivation set to 0.79 in the external test set (117). Critically, no AI-driven nutritional intervention models specifically tailored to DFU populations have been developed to date. The absence of standardized, multi-center, prospectively collected datasets that integrate clinical, imaging, nutritional, and metabolic data makes both the training and robust external validation of such models impossible at present. To overcome this limitation, future research initiatives must prioritize the establishment of these multimodal registries and adhere strictly to reporting guidelines such as TRIPOD+AI to ensure transparent and reproducible model development.

### 6.6 Technical and methodological challenges of multi-modal data integration

Even when data availability, method validity, economic feasibility, and model validation are addressed, synthesizing heterogeneous data types—including clinical, imaging, nutritional, and metabolic inputs—presents substantial technical and methodological hurdles. Heterogeneity in data formats, non-random data missingness, temporal irregularity, and a pervasive lack of interoperability between electronic health record systems remain major barriers to clinical deployment. Advanced machine learning architectures—such as multi-modal fusion networks, transformers, and graph neural networks—offer promising avenues for synthesizing structured and unstructured data into unified risk models (118, 119). Beyond current applications in medical imaging, genomics, and electronic health records, the adoption of federated learning frameworks holds distinct promise for multi-center DFU research. By enabling collaborative model training across diverse institutions without compromising patient privacy or moving raw data across borders, federated learning can enrich models with varied nutritional and metabolic profiles from distinct geographic populations. Addressing this nutritional data gap is not merely a technical refinement but a conceptual imperative for the next generation of AI tools in DFU care. Only by embracing a truly multi-modal, data-driven approach—one that integrates clinical, imaging, metabolic, and nutritional dimensions—can AI deliver precision, equity, and efficacy in diabetic foot management.

## 7 Prospects

### 7.1 Trends in technological innovation

Emerging AI technologies, such as reinforcement learning and generative adversarial networks (GANs), show significant potential for advancing the management and treatment of DFUs by enhancing diagnostic accuracy, improving treatment protocols, and streamlining personalized patient care. Reinforcement learning, a type of machine learning in which an agent learns to make decisions by receiving rewards or penalties based on its actions, can optimize treatment strategies for DFUs over time by analyzing longitudinal patient data, including wound characteristics, treatment responses, and healing rates. Predictive modeling suggests that reinforcement learning models can be leveraged to determine the optimal combinations of dressings, medications, and clinical interventions for specific patient profiles. This approach leads to more tailored care pathways and minimizes the risk of severe complications, such as secondary infections or lower-extremity amputations (50).

GANs can be used to improve the accuracy of image analysis and the classification of DFUs. GANs consist of two interconnected neural networks—the generator and the discriminator—that work adversarially to improve the quality of generated data. In DFU clinical research, GANs can be employed to generate synthetic images of various ulcer types to augment training datasets for machine learning models. This application is particularly valuable in scenarios where obtaining a large volume of annotated clinical images is restricted by institutional privacy or scarcity. By training on these expanded, diverse datasets, AI models can achieve higher accuracy in ulcer classification and wound healing velocity prediction (52). Moreover, GANs have the potential to assist in the development of advanced diagnostic tools that comprehensively analyze plantar and dorsal foot images to detect early signs of ulceration, providing clinicians with real-time assessments of tissue viability. This real-time capability enables timely clinical interventions that can halt ulcer progression and reduce the incidence of severe secondary complications (4, 48). For instance, the AFSegGAN model, which employs a conditional GAN architecture, was designed to automate wound segmentation and estimate the morphological parameters of DFUs; this model has been reported to demonstrate superior edge-detection performance compared to traditional architectures, achieving high structural similarity and accuracy metrics (120). By employing techniques such as image-to-image translation, GANs improve the spatial resolution and clarity of raw wound photographs. This enhancement enables healthcare providers to precisely assess the severity and tissue characteristics of DFUs, leading to objective clinical evaluations and better-informed treatment strategies (120). Beyond segmentation and image enhancement, GAN frameworks could potentially aid in predicting the risk of future ulceration, localized infections, or ischemic tissue changes by analyzing deep features extracted from the wound bed—a capability crucial to preventive management for mitigating the long-term morbidity associated with diabetic foot pathology (52, 121). Integrating GAN-generated synthetic data with CNNs to form a unified diagnostic system further may enhance the accuracy of DFU classification into distinct clinical categories, such as “ulcer,” “infection,” “normal,” and “gangrene.” This integration provides a more nuanced stratification of the patient’s immediate wound status, thereby optimizing the design of tailored treatment plans (3). Ultimately, by optimizing image quality, expanding sparse datasets through targeted data augmentation, and enhancing multi-class predictive capabilities, GANs support more effective management strategies that improve patient outcomes and alleviate the heavy healthcare costs associated with chronic diabetic foot complications.

The integration of AI technologies in the management of DFUs can be extended to patient education and self-management. AI-driven applications provide personalized recommendations for foot care, dietary adjustments, and glycemic control that are crucial for DFU prevention. By utilizing reinforcement learning, these systems can adapt their recommendations based on user feedback and longitudinal health outcomes, promoting better adherence to self-care protocols (100). Furthermore, the economic implications of implementing AI systems in this clinical domain are noteworthy. By improving diagnostic accuracy and treatment efficacy, AI interventions potentially reduce the substantial healthcare expenditures associated with advanced DFU complications, such as prolonged hospitalizations and lower-extremity surgical interventions, particularly in regions experiencing a high prevalence of diabetes (54). Ultimately, deploying emerging AI methodologies—such as reinforcement learning and GANs—presents promising prospects for enhancing diagnostic capabilities, optimizing treatment plans, and improving patient outcomes, ultimately contributing to better management of this complex and challenging condition. As research evolves, integrating these computational models into standard clinical workflows may revolutionize the approach to diabetic foot care, leading to more effective prevention and treatment strategies.

### 7.2 The integration of other cutting-edge technologies, such as 3D bioprinting and gene therapy, in the management of DFUs

Newly developed cutting-edge technologies, such as 3D bioprinting and gene therapy, are being explored to enhance the diagnosis and treatment of DFUs. 3D bioprinting, a revolutionary technique in the field of regenerative medicine, particularly for wound healing, allows the creation of customized tissue scaffolds that can mimic the natural extracellular matrix of human tissue. This technique can produce skin substitutes that meet the specific needs of patients and address the unique characteristics of their wounds, such as size, depth, and tissue type, through the use of biocompatible materials and living cells. The application of 3D bioprinting not only enhances the healing process but also reduces the risk of infection and improves overall DFU outcomes by providing a more effective and personalized treatment approach (122). Gene therapy, another innovative technology employed to deliver therapeutic genes to the wound site to promote healing and tissue regeneration, is being explored for the treatment of DFUs. Regarding the impaired blood flow and delayed wound healing associated with DFUs, genes that encode growth factors can be introduced to stimulate angiogenesis, improve healing rates, and reduce the incidence of complications (122). The integration of these cutting-edge technologies into the management of DFUs indicates that clinical paradigms are shifting toward personalized medicine. The complexity of DFUs necessitates a multifaceted approach that combines various treatment regimens. The combination of 3D bioprinting with gene therapy synergistically enhances healing by not only meeting the immediate requirements of the wound bed but also targeting the mechanisms underlying delayed healing, such as poor vascularization and chronic inflammation (122). The integration of 3D bioprinting and gene therapy into the management of DFUs offers innovative solutions to the challenges posed by this condition, potentially leading to reduced rates of amputation, improved overall outcomes, and enhanced quality of life for patients.

### 7.3 Future directions for the diagnosis and treatment of DFUs: multidisciplinary collaboration

Given their complex characteristics, the future directions for the diagnosis and treatment of DFUs lean toward the development of multidisciplinary approaches that require collaboration among various healthcare professionals, including endocrinologists, vascular surgeons, orthopedic specialists, diabetes educators, and nursing staff. The integration of advanced technologies and evidence-based guidelines into clinical practice represents a promising avenue in DFU care. The International Working Group on the Diabetic Foot (IWGDF) has developed comprehensive guidelines that emphasize the importance of a structured approach to the prevention and management of DFUs. These guidelines provide a framework for clinicians, ensuring that all aspects of patient care are addressed, from initial assessment to ongoing management and education (123). Moreover, the integration of MDTs has shown significant benefits in reducing major amputation rates and improving patient outcomes. This success is largely due to the coordinated care that MDTs provide, which allows for timely interventions and comprehensive management strategies tailored to individual patient needs (123). The establishment of clear treatment workflows and referral pathways within these teams streamlines patient care and enhances communication among specialists (123). While the costs associated with the treatment of diabetic foot complications can be substantial, implementing a multidisciplinary approach reduces hospital stays and the necessity for surgical interventions, thereby lowering overall healthcare costs (124). Other critical components of a multidisciplinary approach, such as regular monitoring and patient education on foot care, can also significantly reduce the incidence of DFUs (125). Further advancements in technology, such as telemedicine and remote monitoring, support ongoing communication between patients and healthcare providers, allowing for the early detection of potential complications and timely interventions (126). Additionally, interdisciplinary education for family caregivers empowers them to support patients in improving self-care, effectively reducing the risk of ulcer development (126). Novel therapeutic approaches, such as bioengineered skin substitutes and advanced wound dressings, are expected to continue providing new options for promoting chronic wound healing (127, 128). Integrating these innovations into a multidisciplinary framework will be crucial for optimizing treatment strategies. This AI-enabled precision nutrition framework remains conceptual and not yet clinically validated, requiring prospective studies to establish safety and efficacy before any clinical deployment. Future DFU diagnosis and management will likely rely on a more collaborative and technology-driven approach; emphasizing multidisciplinary collaboration enhances the quality of care, improves patient outcomes, and mitigates healthcare expenditures associated with diabetic foot complications.

## 8 Conclusion

The convergence of AI and nutritional science represents a promising but nascent area of investigation in DFU management. Nutritional status—quantified through validated tools including the PNI, CONUT score, and micronutrient biomarkers—constitutes an independent determinant of DFU pathogenesis, healing trajectories, and amputation risk. However, this evidence base is derived predominantly from observational studies and retrospective cohorts, with very low certainty of evidence for nutritional interventions as assessed by the GRADE framework. While existing machine learning models have demonstrated proficiency in wound classification and risk stratification using clinical and imaging data, the lack of data regarding dynamic nutritional parameters—dietary intake patterns, body composition metrics, continuous glycemic profiles, and comprehensive biomarker panels—constrains the holistic understanding of DFU pathophysiology and limits precision intervention design.

## Glossary

DFUs: diabetic foot ulcers
AI: artificial intelligence
NeuroQoL: Neuropathy- and Foot Ulcer-Specific Quality of Life Instrument
PVD: Peripheral vascular disease
COVID-19: Corona Virus Disease 2019
MRSA: Methicillin-resistant Staphylococcus aureu
HV: Hallux valgus
DM: Diabetes mellitus
DPN: Determinants of peripheral neuropathy
PAD: Peripheral arterial disease
DKD: Diabetic kidney disease
CKD: Chronic kidney disease
CMM: Cutaneous malignant melanoma
NHANES: National Health and Nutrition Examination Survey
BSL: Blood sugar level
DFEP: Diabetic Foot Education Program
AN: Atonomic neuropathy
ICD-9-CM: International Classification of Diseases, Ninth Revision, Clinical Modification
CNNs: Convolutional neural networks
CKBs: Class knowledge banks
DRNN: Deep recurrent neural network
SOC: Standard of care
RFTM: Remote foot temperature monitoring
HDL: High-density lipoprotein
NLR: Neutrophil to lymphocyte ratio
GANs: Generative adversarial networks
IWGDF: The International Working Group on the Diabetic Foot
MDTs: Multidisciplinary teams

## References

[1] Pandey G Kolipaka T Srinivasarao DA Abraham N Jain A Srivastava S. Navigating the complexities of diabetic foot ulcers: from pathophysiology to advanced treatment strategies. J Drug Delivery Sci Technol. (2025) 107:106852. doi: 10.1016/j.jddst.2025.106852

[2] Hazenberg CEVB Aan De Stegge WB Van Baal SG Moll FL Bus SA. Telehealth and telemedicine applications for the diabetic foot: a systematic review. Diabetes Metabolism Res. (2020) 36:e3247. doi: 10.1002/dmrr.3247, 31808288 PMC7079242

[3] Wang Z Tan X Xue Y Xiao C Yue K Lin K . Smart diabetic foot ulcer scoring system. Sci Rep. (2024) 14:11588. doi: 10.1038/s41598-024-62076-1, 38773207 PMC11109117

[4] Sathya Preiya V Kumar VDA. Deep learning-based classification and feature extraction for predicting pathogenesis of foot ulcers in patients with diabetes. Diagnostics. (2023) 13:1983. doi: 10.3390/diagnostics13121983, 37370878 PMC10297122

[5] Vu GT Tran BX McIntyre RS Pham HQ Phan HT Ha GH . Modeling the research landscapes of artificial intelligence applications in diabetes (GAPRESEARCH). IJERPH. (2020) 17:1982. doi: 10.3390/ijerph17061982, 32192211 PMC7143845

[6] Bhuiyan MNI Saha BK Miah MAS. Artificial intelligence-enabled Tribiotic strategies for gut microbiota modulation in healthy aging and personalized nutrition. Adv Gut Microb Res. (2026) 2026:5517462. doi: 10.1155/agm3/5517462

[7] Coşkun B Ayhan M Ulusoy S. Relationship between prognostic nutritional index and amputation in patients with diabetic foot ulcer. Diagnostics. (2024) 14:738. doi: 10.3390/diagnostics14070738, 38611651 PMC11011454

[8] Can B Kahvecioglu ED Tas IE Kurtuncuoglu SN. Association between prognostic nutritional index and major amputation in patients with diabetic foot ulcers. Haseki. (2026) 64:122–8. doi: 10.4274/haseki.galenos.2026.96967

[9] Oda Y Ikura K Babazono T. Nutritional assessment using the prognostic nutritional index (PNI) and controlling nutritional status (CONUT) score predicts wound healing in patients with diabetic foot ulcers. JED. (2021) 8:1–7. doi: 10.15226/2374-6890/8/1/001151

[10] Nakamura H Makiguchi T Yamada Y Tsunoda A Tomaru N Yokoo S. Evaluation of CONUT score and serum zinc levels in patients with diabetic foot ulcers. The. Int J Lower Extremity Wounds. (2025):15347346251326247. doi: 10.1177/15347346251326247, 40080868

[11] Shi H. Correlation between controlling nutritional status scores and amputation risks in patients with diabetic foot ulcers. J Sichuan Univ. (2022) 53:993–7. doi: 10.12182/20221160209, 36443040 PMC10408985

[12] Karakousis ND Pyrgioti EE Georgakopoulos PN Apergi K Popovic DS Papanas N. Zinc levels and diabetic foot ulcers: a Mini review. Int J Low Extrem Wounds. (2023) 25:276–9. doi: 10.1177/15347346231214209, 37941343

[13] Valayatham VM McGill M Bolton T Nube V Twigg S. PO372 lower serum zinc levels are associated with delayed wound healing in people with diabetic foot ulcers. Diabetes Res Clin Pract. (2014) 106:S236–7. doi: 10.1016/S0168-8227(14)70666-7

[14] Tang W Chen D Chen L Liu G Sun S Wang C . The correlation between serum vitamin D status and the occurrence of diabetic foot ulcers: a comprehensive systematic review and meta-analysis. Sci Rep. (2024) 14:21932. doi: 10.1038/s41598-024-73133-0, 39304728 PMC11415517

[15] Liu L Zhang F Jamali M Guimarães NS Radkhah N Jamilian P . The role of vitamin D in diabetic foot ulcer; an umbrella review of meta-analyses. Front Nutr. (2024) 11:1454779. doi: 10.3389/fnut.2024.1454779, 39444578 PMC11497990

[16] Apergi K Dimosthenopoulos C Papanas N. The role of nutrients and diet characteristics in the Management of Diabetic Foot Ulcers: a systematic review. Int J Low Extrem Wounds. (2025) 24:525–41. doi: 10.1177/15347346231153531, 36734085

[17] Yan C Wang S Yang Y Zhao L Zhang J Wang Y . The efficacy of diabetic foot treatment in a “TOSF” pattern: a five-year retrospective study. DMSO. (2024) 17:1923–39. doi: 10.2147/DMSO.S461112, 38711674 PMC11073528

[18] Donnelly HR Collins CE Clarke ED Morrissey PI Gilbertson-Viljevac N Leigh L . Effectiveness of dietary interventions in individuals with diabetes for preventing and healing chronic wounds; a systematic review with meta-analysis. Diabet Med. (2025) 42:e70100. doi: 10.1111/dme.70100, 40629913 PMC12352720

[19] Hulshagen Y Terwingen J Frings D Verrijken A Van Dessel K Van Gils C . Evolution of muscle mass and strength in patients admitted for a diabetic foot ulcer. Acta Clin Belg. (2025) 80:123–34. doi: 10.1080/17843286.2025.2543334, 40838957

[20] Zheng Z Cao B Ke J Zhao D. The effect of long-term glycemic burden on the incidence of diabetic foot ulcers: a retrospective study. J Diabetes Complicat. (2025) 39:108901. doi: 10.1016/j.jdiacomp.2024.108901 39693944

[21] Dutta A Bhansali A Rastogi A. Early and intensive Glycemic control for diabetic foot ulcer healing: a prospective observational nested cohort study. Int J Low Extrem Wounds. (2023) 22:578–87. doi: 10.1177/15347346211033458, 34279130

[22] Caruso P Scappaticcio L Gicchino M Castaldo F Barrasso M Carbone C . Short-term glucose variability as a determinant of the healing rate of diabetic foot ulcer: a retrospective study. Diabetes Metab Syndr Clin Res Rev. (2024) 18:102990. doi: 10.1016/j.dsx.2024.102990 38508037

[23] Thomason G Gooday C Nunney I Dhatariya K. The association of HbA1c variability with 12 week and 12 month outcomes on diabetes related foot ulcer healing. Diabetes Ther. (2024) 15:2223–32. doi: 10.1007/s13300-024-01640-4, 39153153 PMC11411040

[24] Srinivasan R Sinha A Tentolouris N Jude EB. Impact of continuous glucose monitoring on Glycemic control in type 2 patients with diabetic foot ulcers: a pilot study. Int J Low Extrem Wounds. (2025):15347346251369622. doi: 10.1177/15347346251369622, 40831335

[25] Ortiz-Zúñiga ÁM Simó-Servat O Samaniego J Cuadra-Espinilla F Sánchez M Simó R . Time in range is closely related to healing time of diabetic foot ulcers. Wound Repair Regeneration. (2025) 33:e70052. doi: 10.1111/wrr.70052, 40512319

[26] Dubský M Jirkovská A Bem R Fejfarová V Skibová J Schaper NC . Risk factors for recurrence of diabetic foot ulcers: prospective follow-up analysis in the Eurodiale subgroup. Int Wound J. (2013) 10:555–61. doi: 10.1111/j.1742-481X.2012.01022.x, 22712631 PMC7950559

[27] Iwase M Fujii H Nakamura U Ohkuma T Ide H Jodai-Kitamura T . Incidence of diabetic foot ulcer in Japanese patients with type 2 diabetes mellitus: the Fukuoka diabetes registry. Diabetes Res Clin Pract. (2018) 137:183–9. doi: 10.1016/j.diabres.2018.01.020, 29382584

[28] Guo Q Ying G Jing O Zhang Y Liu Y Deng M . Influencing factors for the recurrence of diabetic foot ulcers: a meta-analysis. Int Wound J. (2023) 20:1762–75. doi: 10.1111/iwj.14017, 36385501 PMC10088840

[29] Hulshof CM Van Netten JJ Pijnappels M Bus SA. The role of foot-loading factors and their associations with ulcer development and ulcer healing in people with diabetes: a systematic review. JCM. (2020) 9:3591. doi: 10.3390/jcm9113591, 33171726 PMC7694972

[30] Bhuiyan MNI Saha BK Satter MA. Harnessing artificial intelligence and precision diets for brain health and cognitive resilience. J Nutr. (2025) 155:3179–90. doi: 10.1016/j.tjnut.2025.08.007, 40812476

[31] Eneroth M Larsson J Oscarsson C Apelqvist J. Nutritional supplementation for diabetic foot ulcers: the first RCT. J Wound Care. (2004) 13:230–4. doi: 10.12968/jowc.2004.13.6.26627, 15214141

[32] Armstrong DG Hanft JR Driver VR Smith APS Lazaro-Martinez JL Reyzelman AM . Effect of oral nutritional supplementation on wound healing in diabetic foot ulcers: a prospective randomized controlled trial. Diabet Med. (2014) 31:1069–77. doi: 10.1111/dme.12509, 24867069 PMC4232867

[33] Momen-Heravi M Barahimi E Razzaghi R Bahmani F Gilasi HR Asemi Z. The effects of zinc supplementation on wound healing and metabolic status in patients with diabetic foot ulcer: a randomized, double-blind, placebo-controlled trial. Wound Repair Regeneration. (2017) 25:512–20. doi: 10.1111/wrr.12537, 28395131

[34] Yeşilyurt M Yüksel S Yosunkaya A. The effect of evidence-based skin care and hydrocolloid dressing in the prevention of nasogastric tube-related pressure injury: a randomized controlled clinical trial. J Tissue Viability. (2024) 33:889–94. doi: 10.1016/j.jtv.2024.09.001, 39289093

[35] Xu X Wang Y Long Y Cheng Y. Chronic constipation and gut microbiota: current research insights and therapeutic implications. Postgrad Med J. (2024) 100:890–7. doi: 10.1093/postmj/qgae112 39237119

[36] Batarbekova S Zhunussova D Derbissalina G Bekbergenova Z Maksimova N Umbetzhanova A . Micronutrient status of patients with diabetic foot: a systematic review. Asia Pac J Clin Nutr. (2025) 34:487–501. doi: 10.6133/apjcn.202508_34(4).0001, 40738717 PMC12310431

[37] Basiri R Spicer MT Levenson CW Ormsbee MJ Ledermann T Arjmandi BH. Nutritional supplementation concurrent with nutrition education accelerates the wound healing process in patients with diabetic foot ulcers. Biomedicine. (2020) 8:263. doi: 10.3390/biomedicines8080263, 32756299 PMC7460445

[38] Basiri R Spicer M Levenson C Ledermann T Akhavan N Arjmandi B. Improving dietary intake of essential nutrients Can ameliorate inflammation in patients with diabetic foot ulcers. Nutrients. (2022) 14:2393. doi: 10.3390/nu14122393, 35745123 PMC9228459

[39] Yu X Liu X Li H. Nutrient metabolism and complications of type 2 diabetes mellitus: implications for rehabilitation and precision care. Front Nutr. (2025) 12:1699259. doi: 10.3389/fnut.2025.1699259, 41190158 PMC12580103

[40] Abuhay HW Yenit MK Wolde HF. Incidence and predictor of diabetic foot ulcer and its association with change in fasting blood sugar among diabetes mellitus patients at referral hospitals in Northwest Ethiopia, 2021. PLoS One. (2022) 17:e0274754. doi: 10.1371/journal.pone.0274754, 36227947 PMC9560537

[41] Vahwere BM Ssebuufu R Namatovu A Kyamanywa P Ntulume I Mugwano I . Factors associated with severity and anatomical distribution of diabetic foot ulcer in Uganda: a multicenter cross-sectional study. BMC Public Health. (2023) 23:463. doi: 10.1186/s12889-023-15383-7, 36899359 PMC9999659

[42] Ullas A Adhikari Mr P Leena K Sasikumar S. Understanding the dynamic relationship of diabetes distress and Glycemic indicators in foot ulcer patients: a correlative study. Cureus. (2024) 16:e57328. doi: 10.7759/cureus.57328, 38690484 PMC11060392

[43] Hasan R Firwana B Elraiyah T Domecq JP Prutsky G Nabhan M . A systematic review and meta-analysis of glycemic control for the prevention of diabetic foot syndrome. J Vasc Surg. (2016) 63:22S–28S.e2. doi: 10.1016/j.jvs.2015.10.005, 26804364

[44] Boyko EJ Zelnick LR Braffett BH Pop-Busui R Cowie CC Lorenzi GM . Risk of foot ulcer and lower-extremity amputation among participants in the diabetes control and complications trial/epidemiology of diabetes interventions and complications study. Diabetes Care. (2022) 45:357–364. doi: 10.2337/dc21-1816 35007329 PMC8914413

[45] Schaper NC Van Netten JJ Apelqvist J Bus SA Fitridge R Game F . Practical guidelines on the prevention and management of diabetes-related foot disease (IWGDF 2023 update). Diabetes Metabolism Res. (2024) 40:e3657. doi: 10.1002/dmrr.3657, 37243927

[46] The Chinese Burn Association, the Yangtze River Delta Integrated Diabetic Foot Alliance, and the Editorial Committee of the Chinese Journal of Burns and Wound Repair Luo G Liu Y Wang A. Practical guidelines for the prevention and management of diabetic foot disease in China. Burns Trauma. (2025) 13:tkaf064. doi: 10.1093/burnst/tkaf064, 41355887 PMC12680013

[47] Schaper NC Van Netten JJ Apelqvist J Bus SA Hinchliffe RJ Lipsky BA . Practical guidelines on the prevention and management of diabetic foot disease (IWGDF 2019 update). Diabetes Metabolism Res. (2020) 36:e3266. doi: 10.1002/dmrr.3266, 32176447

[48] Parveen K Hussain MA Anwar S Elagib HM Kausar MA. Comprehensive review on diabetic foot ulcers and neuropathy: treatment, prevention and management. World. J Diabetes. (2025) 16:14–29. doi: 10.4239/wjd.v16.i3.100329, 40093290 PMC11885961

[49] Kosaji D Awad MI Katmah R Jelinek HF Domingues MF Baguneid M . Diabetic foot prevention, assessment, and management using innovative smart wearable technology: a systematic review. J NeuroEngineering Rehabil. (2025) 22:168. doi: 10.1186/s12984-025-01695-9, 40682082 PMC12273060

[50] Huang H-N Zhang T Yang C-T Sheen Y-J Chen H-M Chen C-J . Image segmentation using transfer learning and fast R-CNN for diabetic foot wound treatments. Front Public Health. (2022) 10:969846. doi: 10.3389/fpubh.2022.969846, 36203688 PMC9530356

[51] Khosa I Raza A Anjum M Ahmad W Shahab S. Automatic diabetic foot ulcer recognition using multi-level thermographic image data. Diagnostics. (2023) 13:2637. doi: 10.3390/diagnostics13162637, 37627896 PMC10453276

[52] Mousa KM Mousa FA Mohamed HS Elsawy MM. Prediction of foot ulcers using artificial intelligence for diabetic patients at Cairo University Hospital. Egypt SAGE Open Nurs. (2023) 9:23779608231185873. doi: 10.1177/23779608231185873, 37435577 PMC10331222

[53] Xie P Li Y Deng B Du C Rui S Deng W . An explainable machine learning model for predicting in-hospital amputation rate of patients with diabetic foot ulcer. Int Wound J. (2022) 19:910–8. doi: 10.1111/iwj.13691, 34520110 PMC9013600

[54] Wang S Wang J Zhu MX Tan Q. Machine learning for the prediction of minor amputation in University of Texas grade 3 diabetic foot ulcers. PLoS One. (2022) 17:e0278445. doi: 10.1371/journal.pone.0278445, 36472981 PMC9725167

[55] Stefanopoulos S Ayoub S Qiu Q Ren G Osman M Nazzal M . Machine learning prediction of diabetic foot ulcers in the inpatient population. Vascular. (2022) 30:1115–23. doi: 10.1177/17085381211040984, 34461765

[56] Nanda R Nath A Patel S Mohapatra E. Machine learning algorithm to evaluate risk factors of diabetic foot ulcers and its severity. Med Biol Eng Comput. (2022) 60:2349–57. doi: 10.1007/s11517-022-02617-w, 35751828

[57] Wu Y Dong D Zhu L Luo Z Liu Y Xie X. Interpretable machine learning models for detecting peripheral neuropathy and lower extremity arterial disease in diabetics: an analysis of critical shared and unique risk factors. BMC Med Inform Decis Mak. (2024) 24:200. doi: 10.1186/s12911-024-02595-z, 39039521 PMC11265186

[58] Bhuiyan MNI Nahid M. Smart nutrition: AI and 3D printing for personalized diets. Food Nutrition. (2025) 1:100032. doi: 10.1016/j.fnutr.2025.100032

[59] Moyen A Rappaport AI Fleurent-Grégoire C Tessier A-J Brazeau A-S Chevalier S. Relative validation of an artificial intelligence–enhanced, image-assisted Mobile app for dietary assessment in adults: randomized crossover study. J Med Internet Res. (2022) 24:e40449. doi: 10.2196/40449, 36409539 PMC9723975

[60] Anjum M Saher R Saeed MN. Optimizing type 2 diabetes management: AI-enhanced time series analysis of continuous glucose monitoring data for personalized dietary intervention. PeerJ Comput Sci. (2024) 10:e1971. doi: 10.7717/peerj-cs.1971, 38686006 PMC11057654

[61] Naja F Taktouk M Matbouli D Khaleel S Maher A Uzun B . Artificial intelligence chatbots for the nutrition management of diabetes and the metabolic syndrome. Eur J Clin Nutr. (2024) 78:887–96. doi: 10.1038/s41430-024-01476-y, 39060542

[62] Jin H Lin Q Lu J Hu C Lu B Jiang N . Evaluating the effectiveness of a generative pretrained transformer-based dietary recommendation system in managing potassium intake for Hemodialysis patients. J Ren Nutr. (2024) 34:539–45. doi: 10.1053/j.jrn.2024.04.001, 38615701

[63] Joshi S Shamanna P Dharmalingam M Vadavi A Keshavamurthy A Shah L . Digital twin-enabled personalized nutrition improves metabolic dysfunction-associated fatty liver disease in type 2 diabetes: results of a 1-year randomized controlled study. Endocr Pract. (2023) 29:960–70. doi: 10.1016/j.eprac.2023.08.016, 37778441

[64] Wang X Sun Z Xue H An R. Artificial intelligence applications to personalized dietary recommendations: a systematic review. Healthcare. (2025) 13:1417. doi: 10.3390/healthcare13121417, 40565444 PMC12193492

[65] Cofre S Sanchez C Quezada-Figueroa G López-Cortés XA. Validity and accuracy of artificial intelligence-based dietary intake assessment methods: a systematic review. Br J Nutr. (2025) 133:1241–53. doi: 10.1017/S0007114525000522, 40207441 PMC12229984

[66] Sosa-Holwerda A Park O-H Albracht-Schulte K Niraula S Thompson L Oldewage-Theron W. The role of artificial intelligence in nutrition research: a scoping review. Nutrients. (2024) 16:2066. doi: 10.3390/nu16132066, 38999814 PMC11243505

[67] Serra M Alceste D Jucker N Haupt L Elben S Müller S . Limited validity of an AI-powered app for dietary assessment in females with obesity. npj Digit Med. (2026) 9:357. doi: 10.1038/s41746-026-02536-2, 41845048 PMC13144502

[68] Panayotova GG. Artificial intelligence in nutrition and dietetics: a comprehensive review of current research. Healthcare. (2025) 13:2579. doi: 10.3390/healthcare13202579, 41154258 PMC12563881

[69] Kirk D. Good practices and common pitfalls of machine learning in nutrition research. Proc Nutr Soc. (2026) 85:253–66. doi: 10.1017/S0029665124007638, 39641238

[70] Hieronimus B Lopez-Aguirre M-L Birringer M Podszun M. GenAI in nutritional sciences (GAINS): a systematic review and reporting framework for future research. Nutr Res. (2025) 143:66–77. doi: 10.1016/j.nutres.2025.09.011, 41138629

[71] Molooy Zada MH Pan D Sun G. A comprehensive systematic review of dynamic nutrient profiling for personalized diet planning: Meta-analysis and PRISMA-based evidence synthesis. Foods. (2025) 14:3625. doi: 10.3390/foods14213625, 41227599 PMC12610451

[72] Agrawal K Goktas P Kumar N Leung M-F. Artificial intelligence in personalized nutrition and food manufacturing: a comprehensive review of methods, applications, and future directions. Front Nutr. (2025) 12:1636980. doi: 10.3389/fnut.2025.1636980, 40771216 PMC12325300

[73] Bhuiyan MNI. Integrating nuclear techniques, isotopic tracing, omics, and artificial intelligence in nutritional Systems for Advancing Precision Public Health Nutrition. J Nutr. (2026) 156:101307. doi: 10.1016/j.tjnut.2025.101307, 41482234

[74] Klonoff DC Bergenstal RM Cengiz E Clements MA Espes D Espinoza J . CGM data analysis 2.0: functional data pattern recognition and artificial intelligence applications. J Diabetes Sci Technol. (2025) 19:1515–1527. doi: 10.1177/19322968251353228 40814224 PMC12356821

[75] Das A Pendsey S Abhyankar M Malabade R. Management of Diabetic Foot in an Indian clinical setup: An opinion survey. Cureus. (2020) 12:e8636. doi: 10.7759/cureus.8636, 32685305 PMC7364422

[76] Alavi A Akhoundi H. "Deep subspace analysing for semi-supervised multi-label classification of diabetic foot ulcer". In: Yap MH Cassidy B Kendrick C, editors. Diabetic Foot Ulcers Grand Challenge, Lecture Notes in Computer Science. Cham: Springer International Publishing (2022). p. 109–20.

[77] Xu Y Han K Zhou Y Wu J Xie X Xiang W. Classification of diabetic foot ulcers using class knowledge Banks. Front Bioeng Biotechnol. (2022) 9:811028. doi: 10.3389/fbioe.2021.811028, 35295708 PMC8918844

[78] Bouallal D Douzi H Harba R. Diabetic foot thermal image segmentation using double encoder-ResUnet (DE-ResUnet). J Med Eng Technol. (2022) 46:378–92. doi: 10.1080/03091902.2022.2077997, 35638349

[79] Biswas S Mostafiz R Uddin MS Paul BK. XAI-FusionNet: diabetic foot ulcer detection based on multi-scale feature fusion with explainable artificial intelligence. Heliyon. (2024) 10:e31228. doi: 10.1016/j.heliyon.2024.e31228, 38803883 PMC11129011

[80] Isaac AL Swartz TD Miller ML Short DJ Wilson EA Chaffo JL . Lower resource utilization for patients with healed diabetic foot ulcers during participation in a prevention program with foot temperature monitoring. BMJ Open Diab Res Care. (2020) 8:e001440. doi: 10.1136/bmjdrc-2020-001440, 33055233 PMC7559055

[81] Ghosh A Ray S Garg MK Chowdhury S Mukhopadhyay S. The role of infrared dermal thermometry in the management of neuropathic diabetic foot ulcers. Diabet Med. (2021) 38:e14368. doi: 10.1111/dme.14368, 32743838

[82] Oe M Tsuruoka K Ohashi Y Takehara K Noguchi H Mori T . Prevention of diabetic foot ulcers using a smartphone and mobile thermography: a case study. J Wound Care. (2021) 30:116–9. doi: 10.12968/jowc.2021.30.2.116, 33573481

[83] Brooks E Burns M Ma R Scholten HJ Becker SH. Remote diabetic foot temperature monitoring for early detection of diabetic foot ulcers: a cost-effectiveness analysis. CEOR. (2021) 13:873–81. doi: 10.2147/CEOR.S322424, 34675567 PMC8504713

[84] Golledge J Fernando ME Alahakoon C Lazzarini PA Aan De Stegge WB Van Netten JJ . Efficacy of at home monitoring of foot temperature for risk reduction of diabetes-related foot ulcer: a meta-analysis. Diabetes Metabolism Res. (2022) 38:e3549. doi: 10.1002/dmrr.3549, 35605998 PMC9541448

[85] Qin Q Oe M Nakagami G Kashiwabara K Sugama J Sanada H . The effectiveness of a thermography-driven preventive foot care protocol on the recurrence of diabetic foot ulcers in low-medical resource settings: An open-labeled randomized controlled trial. Int J Nurs Stud. (2023) 146:104571. doi: 10.1016/j.ijnurstu.2023.104571, 37586286

[86] Thakku Sivakumar D Murray B Moore Z Patton D O’Connor T Avsar P. Can thermography predict diabetic foot ulcer risk in patients with diabetes mellitus? A systematic review. J Tissue Viability. (2024) 33:530–41. doi: 10.1016/j.jtv.2024.06.018, 39025743

[87] Monteiro-Soares M Boyko EJ Jeffcoate W Mills JL Russell D Morbach S . Diabetic foot ulcer classifications: a critical review. Diabetes Metabolism Res. (2020) 36:e3272. doi: 10.1002/dmrr.3272, 32176449

[88] Monteiro-Soares M Hamilton EJ Russell DA Srisawasdi G Boyko EJ Mills JL . Classification of foot ulcers in people with diabetes: a systematic review. Diabetes Metabolism Res. (2024) 40:e3645. doi: 10.1002/dmrr.3645, 37132179

[89] Reddy SS Krishna RS Bendi SK Evangeline BG. Scoring system for assessing risk of amputation in patient’s with diabetic foot. IJAR. (2021) 11:46–8. doi: 10.36106/ijar/4201781

[90] Dörr S Schlecht M Chatzitomaris A Weisser G Lucke-Paulig L Friedl A . Predictive effect of inflammatory response and foot UlcerLocalization on outcome in younger and older individuals with infected DiabeticFoot syndrome. Exp Clin Endocrinol Diabetes. (2021) 129:878–86. doi: 10.1055/a-1149-8989, 32583377

[91] Ahmadi SAY Shirzadegan R Mousavi N Farokhi E Soleimaninejad M Jafarzadeh M. Designing a logistic regression model for a dataset to predict diabetic foot ulcer in diabetic patients: high-density lipoprotein (HDL) cholesterol was the negative predictor. J Diabetes Res. (2021) 2021:1–6. doi: 10.1155/2021/5521493, 33816634 PMC7994070

[92] Serban D Papanas N Dascalu AM Kempler P Raz I Rizvi AA . Significance of neutrophil to lymphocyte ratio (NLR) and platelet lymphocyte ratio (PLR) in diabetic foot ulcer and potential new therapeutic targets. Int J Low Extrem Wounds. (2024) 23:205–16. doi: 10.1177/15347346211057742, 34791913

[93] Kaka AS Landsteiner A Ensrud KE Logan B Sowerby C Ullman K . Risk prediction models for diabetic foot ulcer development or amputation: a review of reviews. J Foot Ankle Res. (2023) 16:13. doi: 10.1186/s13047-023-00610-6, 36922851 PMC10018902

[94] Lee DW Kwak SH Kim JH Choi HJ. Prediction of diabetic foot amputation using newly revised DIRECT coding system: comparison of accuracy with that of five existing classification systems. Int Wound J. (2023) 20:359–71. doi: 10.1111/iwj.13884, 35811359 PMC9885474

[95] Lavery LA Petersen BJ Linders DR Bloom JD Rothenberg GM Armstrong DG. Unilateral remote temperature monitoring to predict future ulceration for the diabetic foot in remission. BMJ Open Diab Res Care. (2019) 7:e000696. doi: 10.1136/bmjdrc-2019-000696 31423317 PMC6688693

[96] Dmitriyeva M Kozhakhmetov SK Turebayev DK Urazova SN Omarov TM Igissinov NS . Monitoring and prevention the risk of diabetic foot ulcer infection during coronavirus Disease-19 pandemic: a narrative review and perspective algorithm. Open Access Maced J Med Sci. (2021) 9:577–82. doi: 10.3889/oamjms.2021.6135

[97] Najafi B Mishra R. Harnessing digital health technologies to remotely manage diabetic foot syndrome: a narrative review. Medicina. (2021) 57:377. doi: 10.3390/medicina57040377, 33919683 PMC8069817

[98] Kong LY Ramirez-GarciaLuna JL Fraser RDJ Wang SC. A 57-year-old Man with type 1 diabetes mellitus and a chronic foot ulcer successfully managed with a remote patient-facing wound care smartphone application. Am J Case Rep. (2021) 22:e933879. doi: 10.12659/AJCR.933879, 34910717 PMC8689370

[99] Littman AJ Timmons AK Korpak A Chan KCG Jones KT Shirley S . Evaluation of the effectiveness of remote foot temperature monitoring for prevention of amputation in a large integrated health care system. Diabetes Care. (2023) 46:1464–8. doi: 10.2337/dc22-1492, 37319007

[100] Demirkol D Erol ÇS Tannier X Özcan T Aktaş Ş. Prediction of amputation risk of patients with diabetic foot using classification algorithms: a clinical study from a tertiary center. Int Wound J. (2024) 21:e14556. doi: 10.1111/iwj.14556, 38272802 PMC10789580

[101] Van Netten JJ Sacco ICN Lavery LA Monteiro-Soares M Rasmussen A Raspovic A . Treatment of modifiable risk factors for foot ulceration in persons with diabetes: a systematic review. Diabetes Metabolism Res. (2020) 36:e3271. doi: 10.1002/dmrr.3271, 31957306

[102] Peng B Min R Liao Y Yu A. Development of predictive nomograms for clinical use to quantify the risk of amputation in patients with diabetic foot ulcer. J Diabetes Res. (2021) 2021:1–9. doi: 10.1155/2021/6621035, 33511218 PMC7822701

[103] Chappell FM Crawford F Horne M Leese GP Martin A Weller D . Development and validation of a clinical prediction rule for development of diabetic foot ulceration: an analysis of data from five cohort studies. BMJ Open Diab Res Care. (2021) 9:e002150. doi: 10.1136/bmjdrc-2021-002150, 34035053 PMC8154962

[104] Aan De Stegge WB Schut MC Abu-Hanna A Van Baal JG Van Netten JJ Bus SA. Development of a prediction model for foot ulcer recurrence in people with diabetes using easy-to-obtain clinical variables. BMJ Open Diab Res Care. (2021) 9:e002257. doi: 10.1136/bmjdrc-2021-002257, 34301678 PMC8311312

[105] Li J Ying C. A sensitivity indicator screening and intelligent classification method for the diagnosis of T2D-CHD. Front Cardiovasc Med. (2024) 11:1358066. doi: 10.3389/fcvm.2024.1358066, 38720918 PMC11076677

[106] Søndergaard SF Christensen JF Dahl M Drejer M Høgh A. The interplay between patients and healthcare professionals in a cross-sectoral setting in connection with the treatment and care of patients with diabetic foot ulcers: a realistic evaluation. BMC Health Serv Res. (2024) 24:782. doi: 10.1186/s12913-024-11219-1, 38982462 PMC11234555

[107] Bi WL Hosny A Schabath MB Giger ML Birkbak NJ Mehrtash A . Artificial intelligence in cancer imaging: clinical challenges and applications. CA A Cancer J Clinicians. (2019) 69:127–57. doi: 10.3322/caac.21552, 30720861 PMC6403009

[108] Shah NG Seam N Woods CJ Fessler HE Goyal M McAreavey D . A longitudinal regional educational model for pulmonary and critical care fellows emphasizing small group- and simulation-based learning. Annals ATS. (2016) 13:469–74. doi: 10.1513/AnnalsATS.201601-027AR, 26845063 PMC5461995

[109] Ring LM DeBitetto J Cheng JJ Yurasek GK. Virtual reality simulation in interprofessional Pediatric cardiology education. Cureus. (2025) 17:e81181. doi: 10.7759/cureus.81181, 40276444 PMC12021375

[110] Rozera T Pasolli E Segata N Ianiro G. Machine learning and artificial intelligence in the multi-omics approach to gut microbiota. Gastroenterology. (2025) 169:487–501. doi: 10.1053/j.gastro.2025.02.035, 40118220

[111] Sounderajah V Guni A Liu X Collins GS Karthikesalingam A Markar SR . The STARD-AI reporting guideline for diagnostic accuracy studies using artificial intelligence. Nat Med. (2025) 31:3283–9. doi: 10.1038/s41591-025-03953-8, 40954311

[112] Matijevich E Minty E Bray E Bachus C Hajizadeh M Liden B. A multi-faceted digital health solution for monitoring and managing diabetic foot ulcer risk: a case series. Sensors. (2024) 24:2675. doi: 10.3390/s24092675, 38732781 PMC11085305

[113] Chang S Zhang F Chen W Zhou J Nie K Deng C . Outcomes of integrated surgical wound treatment mode based on tibial transverse transport for diabetic foot wound. Front Surg. (2023) 9:1051366. doi: 10.3389/fsurg.2022.1051366, 36726959 PMC9885215

[114] Martin WN Wigmore HK Gregory LC Lum CMY Lasschuit JWJ. Overweight yet undernourished: a common juxtaposition in the specialist diabetes foot service. The. Int J Lower Extremity Wounds. (2025):15347346241310266. doi: 10.1177/15347346241310266, 39873149

[115] Jamil S Mohammadnezhad M Abdulrahim A Muhammad F Khan HTA. Managing diabetes one step at a time in low- and middle-income countries: the promise of wearable devices. Chronic Dis Transl Med. (2025) 11:279–83. doi: 10.1002/cdt3.70018, 41341735 PMC12670968

[116] Gardner A Mitchell B Beckingham W Fasugba O. A point prevalence cross-sectional study of healthcare-associated urinary tract infections in six Australian hospitals. BMJ Open. (2014) 4:e005099. doi: 10.1136/bmjopen-2014-005099, 25079929 PMC4120374

[117] Zhang Y Tian Y Jian Y Wei Z Cai S Zhang G . Development and external validation of machine-learning based models to predict diabetic foot ulcer in diabetes population. Front Endocrinol. (2025) 16:1692917. doi: 10.3389/fendo.2025.1692917, 41473241 PMC12745231

[118] Krones F Marikkar U Parsons G Szmul A Mahdi A. Review of multimodal machine learning approaches in healthcare. Inf Fusion. (2025) 114:102690. doi: 10.1016/j.inffus.2024.102690, 41799790 PMC12961302

[119] Stahlschmidt SR Ulfenborg B Synnergren J. Multimodal deep learning for biomedical data fusion: a review. Brief Bioinform. (2022) 23:bbab569. doi: 10.1093/bib/bbab569, 35089332 PMC8921642

[120] Jishnu P Shreyamsha Kumar BK Jayaraman S. Automatic foot ulcer segmentation using conditional generative adversarial network (AFSegGAN): a wound management system. PLOS Digit Health. (2023) 2:e0000344. doi: 10.1371/journal.pdig.0000344, 37930982 PMC10627472

[121] Fan Z Liu Y Xie H Yang Q Zhang G Zhang P . Analysis of risk factors for foot ulcers in diabetes patients with neurovascular complications. BMC Public Health. (2025) 25:792. doi: 10.1186/s12889-025-21639-1, 40011841 PMC11866585

[122] Couturier A Calissi C Cracowski J-L Sigaudo-Roussel D Khouri C Roustit M. Mouse models of diabetes-related ulcers: a systematic review and network meta-analysis. EBioMedicine. (2023) 98:104856. doi: 10.1016/j.ebiom.2023.104856, 38251464 PMC10755106

[123] Haghverdian JC Noori N Hsu AR. Clinical pathway for the Management of Diabetic Foot Infections in the emergency department. Foot Ankle Orthopaedics. (2023) 8:24730114221148166. doi: 10.1177/24730114221148166, 36644108 PMC9834778

[124] Jodheea-Jutton A Hindocha S Bhaw-Luximon A. Health economics of diabetic foot ulcer and recent trends to accelerate treatment. The Foot. (2022) 52:101909. doi: 10.1016/j.foot.2022.101909 36049265

[125] Mavrogenis AF Megaloikonomos PD Antoniadou T Igoumenou VG Panagopoulos GN Dimopoulos L . Current concepts for the evaluation and management of diabetic foot ulcers. EFORT Open Rev. (2018) 3:513–25. doi: 10.1302/2058-5241.3.180010, 30305936 PMC6174858

[126] Wantonoro W Komarudin K Imania DR Harun S Nguyen TV. The influence of 6-month interdisciplinary accompaniment on family caregivers’ knowledge and self-efficacy regarding diabetic wound care. SAGE Open Nurs. (2023) 9:23779608231167801. doi: 10.1177/23779608231167801, 37050936 PMC10084543

[127] Rehman ZU Khan J Noordin S. Diabetic foot ulcers: contemporary assessment and management. J Pak Med Assoc. (2023) 73:1480–8. doi: 10.47391/JPMA.6634, 37469062

[128] Dawi J Tumanyan K Tomas K Misakyan Y Gargaloyan A Gonzalez E . Diabetic foot ulcers: pathophysiology, immune dysregulation, and emerging therapeutic strategies. Biomedicine. (2025) 13:1076. doi: 10.3390/biomedicines13051076, 40426903 PMC12109115

[129] Sun B Chen Y Man Y Fu Y Lin J Chen Z. Clinical value of neutrophil-to-lymphocyte ratio and prognostic nutritional index on prediction of occurrence and development of diabetic foot-induced sepsis. Front Public Health. (2023) 11:1181880. doi: 10.3389/fpubh.2023.1181880, 38026334 PMC10630165

[130] Vlad L Grosser J Dodenhoff K Peoples A Aguilo-Seara G Molnar J. Examining albumin as a bioindicator of healing capability in patients with diabetic foot ulcers: a retrospective review. Wounds. (2023) 35:e193–6. doi: 10.25270/wnds/23012, 37347595

[131] Gao Y-Q Gao Y-H Xing J-H. Vitamin D supplementation reduces infection rate and promotes wound healing in patients with diabetic foot ulcers. World J Diabetes. (2025) 16:108166. doi: 10.4239/wjd.v16.i8.108166, 40837342 PMC12362425

[132] Lane KL Abusamaan MS Voss BF Thurber EG Al-Hajri N Gopakumar S . Glycemic control and diabetic foot ulcer outcomes: a systematic review and meta-analysis of observational studies. J Diabetes Complicat. (2020) 34:107638. doi: 10.1016/j.jdiacomp.2020.107638, 32527671 PMC7721205

[133] Fan H-Y Chen P-R Kuo Y-T Chang Y-T Chien K-L Lu Y-C . Applying a robust deep learning model to assess the remaining amount of foods with high Glycemic index in Taiwanese hospital diabetic meals: a focus on Rice and congee, with Milk as a low-Glycemic reference. Current Dev Nutr. (2025) 9:107569. doi: 10.1016/j.cdnut.2025.107569, 41282519 PMC12639435

[134] Yuan Z Lao G Liu J Jian Y Ran J Chen C . Relationship between low phase angle and impaired healing of diabetic foot ulcer: a retrospective cohort study. Nutr Metab. (2026). doi: 10.1186/s12986-026-01126-z, 42035147 PMC13262210

[135] Chatelan A Clerc A Fonta P-A. ChatGPT and future artificial intelligence chatbots: what may be the influence on credentialed nutrition and dietetics practitioners? J Acad Nutr Diet. (2023) 123:1525–31. doi: 10.1016/j.jand.2023.08.001, 37544375

[136] Arslan S. Exploring the potential of chat GPT in personalized obesity treatment. Ann Biomed Eng. (2023) 51:1887–8. doi: 10.1007/s10439-023-03227-9, 37145177

[137] Rein M Ben-Yacov O Godneva A Shilo S Zmora N Kolobkov D . Effects of personalized diets by prediction of glycemic responses on glycemic control and metabolic health in newly diagnosed T2DM: a randomized dietary intervention pilot trial. BMC Med. (2022) 20:56. doi: 10.1186/s12916-022-02254-y, 35135549 PMC8826661

[138] Chauhan S Kerr A Keogh B Nolan S Casey R Adelfio A . An artificial-intelligence-discovered functional ingredient, NRT_N0G5IJ, derived from *Pisum sativum*, decreases HbA1c in a prediabetic population. Nutrients. (2021) 13:1635. doi: 10.3390/nu13051635, 34068000 PMC8152294

[139] Guan Z Li H Liu R Cai C Liu Y Li J . Artificial intelligence in diabetes management: advancements, opportunities, and challenges. Cell Reports Med. (2023) 4:101213. doi: 10.1016/j.xcrm.2023.101213, 37788667 PMC10591058

[140] Sheng B Pushpanathan K Guan Z Lim QH Lim ZW Yew SME . Artificial intelligence for diabetes care: current and future prospects. Lancet Diabetes Endocrinol. (2024) 12:569–95. doi: 10.1016/S2213-8587(24)00154-2, 39054035

[141] Romero-Tapiador S Lacruz-Pleguezuelos B Tolosana R Freixer G Daza R Fernández-Díaz CM . AI4FoodDB: a database for personalized e-health nutrition and lifestyle through wearable devices and artificial intelligence. Database. (2023) 2023:baad049. doi: 10.1093/database/baad049, 37465917 PMC10354505

