# Supplementary material for: Precision nutrition in diabetic foot ulcers: multimodal artificial intelligence for personalized metabolic management
Source: Front Nutr. 2026 Jun 17;13:1821103. doi: 10.3389/fnut.2026.1821103 (PMC13319797; doi:10.3389/fnut.2026.1821103)
Supplement: Supplementary file 1 [file Table_1.docx]

| Table 1. Previous Studies on Risk Factors for Diabetic Foot Ulcers (DFUs) | | | | |
| --- | --- | --- | --- | --- |
| Refs. | Main Focus | Key Findings | Methodology | Relevant Conclusions |
| (Sabapathy and Periasamy, 2016) | Diabetic neuropathy | Neuropathy causes sensation loss and foot deformities, contributing to ulcer formation. | Review | Neuropathy is a primary cause of DFUs. |
| (Jude et al., 2001) | Neuropathy severity | Severity of neuropathy is the most important factor associated with DFUs. | Cohort study | Neuropathy severity predicts DFU development and recurrence. |
| (Vileikyte et al., 2003) | Quality of life | Developed NeuroQoL to evaluate the impact of neuropathy on quality of life. | Instrument development | Neuropathy significantly affects quality of life. |
| (Galkowska et al., 2006) | Neurogenic factors | Correlation of neurogenic factors with DFU healing process. | Review | Neurogenic factors influence DFU healing. |
| (Ndip and Jude, 2009) | Neuroischemic DFUs | Emphasized the importance of vascular review in all patients. | Review | Vascular review is crucial for neuroischemic DFUs. |
| (Amin and Doupis, 2016) | Diabetic peripheral neuropathy (DPN) | DPN is a major risk factor for foot ulceration. | Review | DPN significantly increases the risk of DFUs. |
| (Dietrich et al., 2017) | Diabetic foot syndrome | Emphasized the importance of DPN in diabetic foot syndrome. | Review | DPN is a key factor in diabetic foot syndrome. |
| (Zubair and Ahmad, 2019) | Cytokines and wound healing | Changes in cytokine levels can accelerate wound healing. | Review | Regulating cytokine levels can improve DFU healing. |
| (López-Moral et al., 2019) | Therapeutic footwear | Use of therapeutic footwear with a rigid rocker sole in diabetic patients with neuropathy. | Review | Therapeutic footwear can prevent DFUs. |
| (Yuzuguldu et al., 2023) | Multiple risk factors | Highlighted poor glycemic control and vascular disease as critical risk factors. | Review | Comprehensive management of risk factors is essential. |
| (Das et al., 2020) | Peripheral Vascular Disease (PVD) | PVD is a critical risk factor for DFUs. | Review | Early detection and management of PVD are crucial. |
| (Dinoto et al., 2021) | Endovascular procedures | Multilevel revascularization showed promising effects in diabetic COVID-19 patients. | Case study | Endovascular procedures can improve outcomes. |
| (Dama et al., 2023) | Stem cell therapy | Bone marrow-derived mesenchymal stem cells can promote neovascularization and healing. | Review | Stem cell therapy is a potential treatment option. |
| (Kang et al., 2024) | Peripheral arterial disease | Revascularization significantly improved amputation-free survival rates. | Cohort study | Revascularization is effective in managing PVD. |
| (Hu et al., 2024) | Recurrence of DFUs | Peripheral arterial disease is essential to mitigate high long-term recurrence rates. | Cohort study | Managing PVD reduces DFU recurrence. |
| (Barn et al., 2015) | Foot deformities | Structural abnormalities contribute to uneven pressure distribution and ulceration. | Review | Regular foot inspections are crucial. |
| (Song and Chambers, 2025) | Hammertoes and claw toe | High prevalence of foot deformities in diabetic patients. | Review | Foot deformities are significant risk factors. |
| (Zubair and Ahmad, 2019) | Structural abnormalities | Structural abnormalities contribute to DFU development. | Review | Regular foot inspections are essential. |
| (Boulton and Whitehouse, 2000) | Foot care | Regular foot inspections are crucial for early detection of complications. | Review | Early detection prevents severe complications. |
| (Orioli et al., 2020) | Glycemic control | Emphasized the importance of glycemic control in preventing DFUs. | Review | Poor glycemic control increases DFU risk. |
| (Shabhay et al., 2021) | Clinical profiles | Severe consequences of poor glycemic control in DFU patients. | Case study | Poor glycemic control leads to severe complications. |
| (Ouyang et al., 2021) | Risk factors in Type 2 diabetes | Poor glycemic control is associated with DFU development. | Cohort study | Glycemic control is critical in managing DFUs. |
| (Shah et al., 2022) | Wagner's classification | Poor glycemic control increases risks for DFU complications. | Review | Glycemic control is essential for DFU management. |
| (Yin et al., 2023) | Mendelian randomization | Emphasized the significance of glycemic control and foot care. | Review | Glycemic control and foot care are crucial. |
| (Cheng et al., 2021) | Recurrent DFUs | Diabetes duration, callus, and history of vascular intervention are risk factors for recurrence. | Cohort study | Multiple factors contribute to DFU recurrence. |
| (Tan Dat et al., 2023) | Re-ulceration rate | Higher re-ulceration rate in lower extremity amputation patients. | Cohort study | Early diagnosis and care are crucial. |
| (Giurato et al., 2023) | Risk factors for recurrence | Multiple risk factors identified in patients managed by an integrated foot care protocol. | Cohort study | Integrated care is essential for DFU management. |
| (Sharma et al., 2021) | Infection complications | Increased risk of acute admission and amputation due to infection. | Review | Infection significantly impacts DFU outcomes. |
| (Lou et al., 2025) | Infection prevalence | High prevalence of infection in DFU patients. | Review | Infection is a significant factor in DFUs. |
