# Supplementary material for: Precision nutrition in diabetic foot ulcers: multimodal artificial intelligence for personalized metabolic management
Source: Front Nutr. 2026 Jun 17;13:1821103. doi: 10.3389/fnut.2026.1821103 (PMC13319797; doi:10.3389/fnut.2026.1821103)
Supplement: Supplementary file 2 [file Table_2.docx]

| Table 2. Comparative Analysis of Studies on the Prevention of Diabetic Foot Ulcers | | | | |
| --- | --- | --- | --- | --- |
| Refs. | Study Design | Sample Population | Key Interventions/Assessments | Main Findings |
| (Hirpha et al., 2020) | Cross-sectional study | Diabetic patients at Jimma Medical Center’s ambulatory clinic | Self-inspection practices of diabetic patients | Inadequate self-inspection of feet among diabetic patients. Emphasis on the need for clinicians to highlight the importance of foot self-inspection. |
| (Akila et al., 2021) | Cross-sectional study | Diabetic patients in South India | Regular foot screening practices | Importance of early identification of foot ulcer risk through regular screening. |
| (Liu et al., 2020) | Descriptive study | Diabetic patients in Beijing, China | Self-foot examination and professional foot inspection knowledge and behavior scores | Low to moderate levels of foot care knowledge and behavior related to self-foot examination and professional inspection. |
| (Wang et al., 2023) | Cross-sectional study using NHANES 2011-2018 data | Diabetic patients in the United States | Knowledge and behavior regarding proactive foot ulcer examinations | Gaps in knowledge and behavior of diabetic patients in being proactive in foot ulcer examinations. |
| (Ahmed et al., 2020) | Systematic literature review | Various studies on footwear and insole design | Optimizing footwear design and insole features | Importance of footwear and insole design in reducing plantar pressures and preventing DFUs. |
| (Yavuz et al., 2020) | Interventional study | Diabetic patients | Use of temperature- and pressure-regulating insoles | Effectiveness of regulating insoles in preventing DFUs. |
| (Zwaferink et al., 2020) | Efficacy testing | Diabetic patients | Data-driven custom-made footwear | Scientific-based custom footwear design effectively prevents DFUs by relieving plantar pressure. |
| (Bus et al., 2024) | Updated guideline | Healthcare professionals and diabetic patients | Education on proper footwear for moderate-to-high risk patients | Properly-fitted, accommodative, and therapeutic footwear crucial for preventing DFUs. |
| (Moulaei et al., 2019) | Review | General diabetic patient population | Foot hygiene practices | Importance of daily cleaning, drying, and moisturizing feet (excluding between toes) to prevent DFUs. |
| (Tuha et al., 2021) | Knowledge and practice assessment | Diabetic patients at Dessie referral hospital, Ethiopia | Diabetic foot self-care knowledge and practice | Good knowledge and practice of diabetic foot self-care reduce, delay, or prevent complications. |
| (Elkashif et al., 2021) | Self-care practice protocol evaluation | Diabetic foot patients | Implementation of self-care practice protocol | Improved foot care knowledge and self-care practice scores post-protocol intervention. |
| (Adeyemi et al., 2021) | Knowledge, attitude, and practice study | Diabetic patients in Tobago | Foot care practices | Majority of diabetic patients had poor knowledge and inadequate health education regarding DFUs. |
| (Mafusi et al., 2024) | Knowledge assessment | Nurses in South Africa | Knowledge on diabetic foot care guidelines | Majority of nurses had adequate knowledge, but ongoing education was still needed. |
| (Abuhay et al., 2022) | Correlational study | Diabetes mellitus patients | Incidence of DFUs and fasting blood sugar levels | Association between changes in fasting BSL and DFU incidence, emphasizing BSL monitoring. |
| (Vahwere et al., 2023) | Observational study | Patients with DFUs | Severity and anatomical distribution of DFUs | Uncontrolled BSL associated with severe DFUs. |
| (Kilic et al., 2025) | Suggested awareness programs | Diabetic patients | Importance of BSL monitoring under medical supervision | Extensive awareness programs needed to control negative effects of prolonged diabetes duration. |
| (Ullas et al., 2024) | Descriptive correlational study | Patients with DFUs | Relationship between diabetes distress and glycemic indicators | Significant correlation between diabetes distress and glycemic indicators, highlighting comprehensive care needs. |
| (Das et al., 2020) | Structured questionnaire | Physicians in India participating in DFEP | Diabetic foot education programs | Need for education programs to promote awareness and implement foot-care strategies. |
| (Pavithra et al., 2020) | Awareness and practice assessment | Diabetic patients in Southern India | Foot care awareness and practice | Need for health education addressing awareness gaps in diabetic patients. |
| (Miranda et al., 2021) | Update on prevention | Healthcare providers and diabetic patients | Continuous education on DFU prevention | Importance of ongoing education for both patients and healthcare providers. |
| (Yang et al., 2022) | Audit of DFU risk assessment protocols | Various clinical settings | Regular audits of DFU risk assessment protocols | Regular audits help identify barriers to implementation. |
| (Bubun et al., 2023) | Validity and reliability testing | Community setting | Diabetic foot check-up | Potential of community check-ups as early screening tools for DFU risk. |
| (Bouly et al., 2022) | Follow-up study | Post-healing DFU patients | Relation between follow-up and recurrence rate | Multidisciplinary follow-up reduces DFU recurrence risk. |
| (Zia Ur Rehman et al., 2023) | Literature review | Various studies on DFU management | Contemporary assessment and management strategies | Need for ongoing monitoring and aggressive management strategies. |
| (Soria et al., 2023) | Clinical trial | Patients with vascular and neuropathic complications | Cell therapy targeting peripheral arterial disease | Cell therapy as a potential alternative strategy to prevent limb amputations. |
| (Guo et al., 2023) | Systematic review | Various studies on DFU recurrence | Risk factors for DFU recurrence | Diabetic peripheral neuropathy, PVD, and history of vascular intervention significantly correlated to DFU recurrence. |
| (Najafi and Mishra, 2021) | Summary of innovations | Various studies on digital health technologies | Innovations in digital health for DFU prevention | Importance of new technologies in controlling reversible risk factors of DFUs. |
| (Hijriana and Miniharianti, 2021) | Interventional study | Diabetes mellitus Type 2 patients | Continuous physical exercises (e.g., foot massage, joint movement) | Prevention of long-term complications such as diabetic neuropathy and PVD. |
| (Lepesis et al., 2023) | Interventional study | Patients with diabetic peripheral neuropathy and limited joint mobility syndrome | Ankle and 1st metatarsophalangeal joint mobilizations with home stretches | Improved joint mobility and stability. |
| (Van Netten et al., 2020) | Systematic review | Various studies on interventions for DFUs | Effectiveness of custom-made therapeutic footwear | Custom orthotics crucial in preventing DFUs by improving modifiable risk factors. |
| (Matijevich et al., 2024) | Digital health solution application | Diabetic patients | Multi-faceted digital health solution (e.g., custom sensory insoles) | Improved integrative foot care guidelines through digital health solutions. |
| (Jones et al., 2024) | Systematic review | Various studies on offloading devices | Custom-made vs. standard offloading devices | Custom-made devices superior in preventing DFUs. |
| (Kaminski et al., 2022) | Evidence-based guidelines | Diabetic patients at increased risk of DFU | Recommendations on protective footwear | Avoidance of walking barefoot, in socks without shoes, or in inappropriate footwear. |
| (Alshammari et al., 2023) | Review | General diabetic patient population | Importance of following foot care guidelines | Reduction in DFU development through proper foot care practices. |
| (Brenna Santos Batista and Caroline Santana Santos, 2021) | Case report | 71-year-old female patient with a foot ulcer | Nutritional follow-up during hospitalization | Importance of nutritional control in promoting ulcer healing. |
| (Miraj et al., 2023) | Review | Various studies on phytotherapy | Effect of phytotherapy on DFU management | Phytotherapy as a promising strategy for managing diabetic foot complications. |
| (Highton et al., 2024) | Review | Individuals with diabetic foot disease | Non-pharmacological interventions | Potential of nutritional supplementation in reducing cardiovascular disease risk. |
